# Structural insights into the enzymatic breakdown of azomycin-derived antibiotics by 2-nitroimdazole hydrolase (NnhA)

**DOI:** 10.1038/s42003-024-07336-6

**Published:** 2024-12-19

**Authors:** F. Hafna Ahmed, Jian-Wei Liu, Santana Royan, Andrew C. Warden, Lygie Esquirol, Gunjan Pandey, Janet Newman, Colin Scott, Thomas S. Peat

**Affiliations:** 1 Environment, CSIRO, Canberra, ACT 2601 Australia; 2 Advanced Engineering Biology Future Science Platform, CSIRO, Canberra, ACT 2601 Australia; 3 Manufacturing, CSIRO, 343 Royal Parade, Parkville, VIC 3052 Australia; 4 BABS, UNSW, Kensington, NSW 2052 Australia; 5 ARC Centre of Excellence in Synthetic Biology, CSIRO, Canberra, ACT 2601 Australia

**Keywords:** X-ray crystallography, Hydrolases, Antimicrobial resistance

## Abstract

The antibiotic 2-nitroimidazole (2NI) or azomycin, used for treating drug-resistant tuberculosis and imaging tumor hypoxia, requires activation by bacterial nitroreductases for its antibiotic and cytotoxic effect. *Mycobacterium sp. JS330* produces 2-nitroimidazole nitrohydrolase (NnhA) that circumvents 2NI activation, conferring 2NI resistance by hydrolysing it to nitrite and imidazol-2-one (IM2O) instead. This study elucidates NnhA’s structure, catalytic mechanism, and evolutionary background within the guanidino-group modifying enzyme (GME) superfamily, aided by a more soluble protein variant engineered through directed evolution. Despite low sequence similarity and limited occurrence in a few soil-dwelling mycobacteria and Actinomycetota, NnhA maintains the α/β propeller fold characteristic of GME superfamily enzymes and forms an unusual hexameric ring structure formed by a trimer of domain-swapped dimers. The similarity of its active site to arginine deiminases (ADIs) and human dimethylarginine dimethylaminohydrolases (DDAHs), along with molecular dynamics simulations, suggests NnhA’s catalytic mechanism resembles the hydrolysis reactions of these related enzymes.

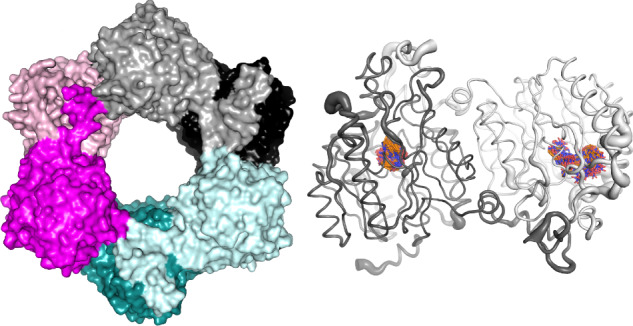

## Abstract (web summary)

Experimentally determined structure of 2-nitroimidazole hydrolase and its comparison with other related enzymes shed light on the mechanism of action of an unusual antibiotic degrading enzyme in soil bacteria.

## Introduction

**Fig. 1:**
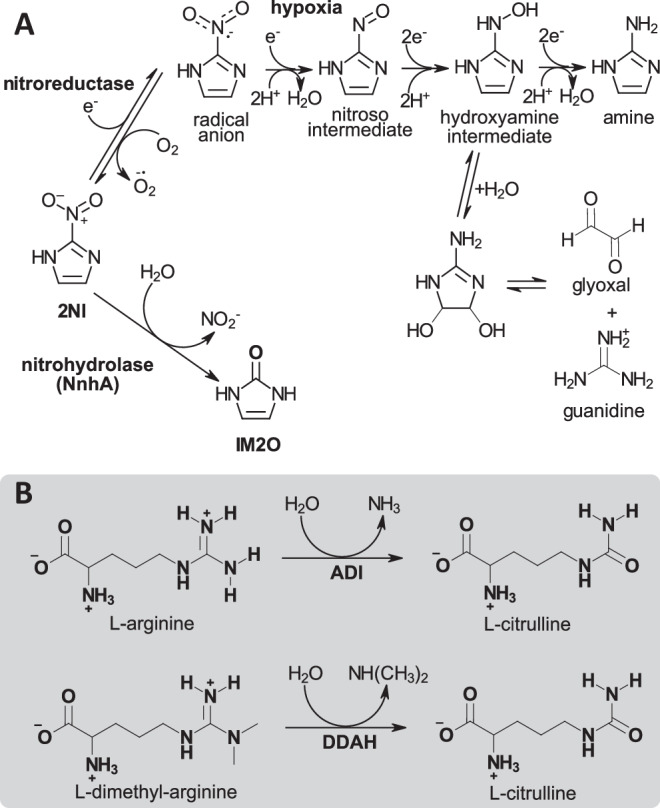
Reaction schemes for NnhA and other GME superfamily enzymes. **A** Comparison of the nitroreductase and nitrohydrolase (NnhA) mediated degradation pathways for 2NI^3,13^. **B** Substrates and products for arginine deiminases (ADIs) and human dimethylarginine dimethylaminohydrolases (DDAHs). For DDAH, only N^G^, N^G^ asymmetric dimethylarginine (ADMA) is shown as a substrate with the resulting di-methylamine product, although N^G^ methylarginine (L-NMMA) is also accepted, producing mono-methylamine instead.

Many antibiotics and antibiotic precursors that are used in human therapies were initially isolated from bacteria. One such naturally occurring antimicrobial is 2-nitroimidazole (2NI, azomycin), produced by soil bacteria of the orders *Actinomycetales* and *Pseudomonales*^1–3^. 2NI is currently used as a treatment for drug-resistant tuberculosis^4^. Its derivatives are clinically used for imaging tumor hypoxia as an indication of chemotherapy resistance^5–7^, and show promising anti-tumor activity under hypoxic conditions^8,9^. Like other nitroimidazoles, 2NI is a prodrug that requires activation by endogenous nitroreductases to a radical anion^8,10–13^. Under hypoxia, this radical anion forms cytotoxic nitroso and hydroxylamine species that are trapped within cells and are further reduced to glyoxal that damages DNA by forming adducts with guanine bases (Fig. 1A).characterization

Resistance to naturally occurring antibiotic compounds is inevitably found in nature as well. In 2011, the soil-dwelling bacterium *Mycobacterium sp. JS330* was observed to grow in the presence of 2NI, and the gene encoding for 2NI nitrohydrolase A (NnhA) was discovered to be essential for bacterial survival in the presence of 2NI^3^. NnhA hydrolyzes the nitro group from 2NI to form nitrite and imidazol-2-one (IM2O)^3^, preventing its reduction to the cytotoxic intermediates and products that elicit the antibiotic or hypoxic effect (Fig. 1A). The presence of NnhA is sufficient to confer 2NI resistance in bacteria, as its heterologous expression in *Escherichia coli* cells makes them resistant to the antibiotic effect of 2NI^3^. Furthermore, *M. sp. JS330* can fully metabolize the IM2O product as well, with growth even when 2NI is provided as the sole carbon source^3^.

The initial characterization of NnhA indicated that, unlike most other known enzymes that degrade nitroaromatic compounds^14^, NnhA is a hydrolase that functions without catalytic metal ions, molecular oxygen, or other cofactors^3^. It also appears to be specific towards 2-nitroimidazoles, with activity seen with 2NI and its synthetic derivative benznidazole, but not with 4-nitroimidazoles, 5-nitroimidazoles, 2,4-nitroimidazoles, or thiazolidines^3^. The closest related functionally characterized proteins to NnhA, with low ~20% sequence identities, are the arginine deiminases (ADIs) and human dimethylarginine dimethylaminohydrolases (DDAHs)^3^. Unlike the nitroaromatic substrate of NnhA, both ADIs and DDAHs catalyze the hydrolysis of guanidino-group containing substrates (Fig. 1B). ADIs catalyze the hydrolysis of L-arginine to citrulline while releasing ammonia^15^, and DDAHs catalyze the hydrolysis of N^G^, N^G^ asymmetric dimethylarginine (ADMA) or N^G^ methylarginine (l-NMMA) to citrulline and di- or mono-methylamine, respectively^16^.

Both ADI and DDAH enzymes are cysteine hydrolases that contain a catalytic triad, with a basic histidine residue stabilized by a glutamic acid residue, and a nucleophilic cysteine residue^17–24^. Their reactions proceed via a covalent intermediate formed between the catalytic cysteine and the substrate, which leads to the elimination of ammonia or mono-/di-methylamine. This is followed by hydrolysis of the cysteine-to-substrate covalent bond by a water molecule activated by the stabilized histidine residue. Despite the difference in substrate preference, it has been suggested that the presence of the same conserved protein domain in NnhA as ADIs and DDAHs may indicate a related reaction mechanism^3^.

Nitrohydrolases are uncommon; to the best of our knowledge, NnhA is currently the only enzyme annotated with this activity^25^. In addition to its ability to degrade 2NI antibiotics, it may be possible to engineer this enzyme to degrade semi-stable compounds with nitro groups, such as those found in many explosive compounds^14^. This would be beneficial to the many decommissioned military bases scattered throughout the world which could then be potentially repurposed post the bioremediation process.

In this work, we have characterized the structure of NnhA and an inactive NnhA mutant using X-ray crystallography. For the structural characterization, we initially used directed evolution to obtain a stable and soluble variant of NnhA that can be readily produced in *E. coli*. By assessing the activity of NnhA variants with active site mutations and performing Molecular Dynamics (MD) simulations with the structure of the soluble variant, we have proposed a potential reaction mechanism for 2NI hydrolysis by NnhA. Along with a sequence and phylogenetic analysis of the relationships between NnhA, ADI, DDAH, and other related proteins, we present a detailed sequence-structure-function characterization of this unusual enzyme.

## Results and discussion

### Sequence similarity of NnhA with other GME superfamily enzymes

Given the previously noted sequence similarity of NnhA to ADI and DDAH enzymes^3^, we first performed a sequence analysis of NnhA (Uniprot Accession: F4ZCI3.1) and its related proteins to inform its structural and function characterization. NnhA resembles proteins of the guanidino-group modifying enzyme (GME) superfamily (Pfam ID: CL0197), which consists of several protein families that share an α/β propeller fold^23,24,26^. This includes arginine deaminases (ADI), human dimethylarginine dimethylaminohydrolases (DDAH), agmatine deiminases, peptidylarginine deiminases (PAD), *N*-succinylarginine dihydrolases (AstB), and eukaryotic translation initiation factor 6 (eIF-6).

From the retrieved sequences, we produced a sequence similarity network (SSN) to cluster closely related proteins that can represent functional groups^27–30^. In this SSN, each node represents a cluster of proteins with >45% sequence similarity, and edges between nodes represent an alignment score that broadly indicates the sequence similarity between the nodes^27^. Increasing the alignment score cut-off for network visualization allows ‘zooming’ into relationships between more closely related proteins.

**Fig. 2:**
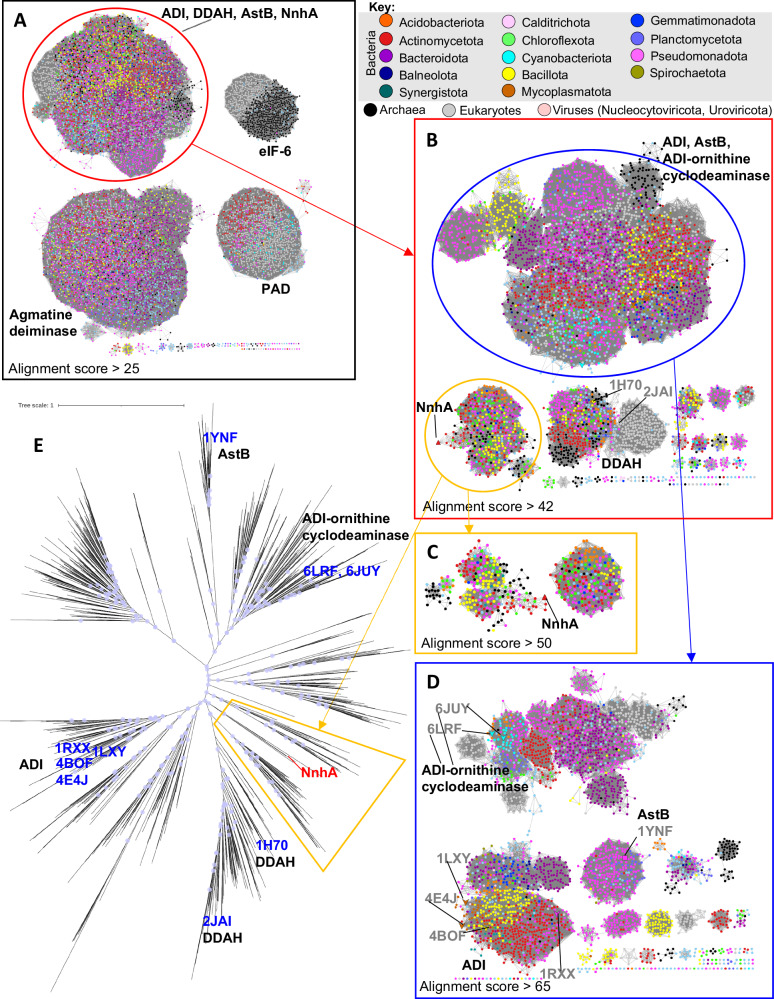
Sequence and phylogenetic analysis of the GME superfamily. **A** SSN of the GME superfamily (Pfam ID: CL0197) at an alignment score cut-off >25, where each node represents proteins with >45% sequence similarity. Edges represent the alignment score calculated by EFI-EST^27^, which approximately represents the BLAST logE value between protein sequences. Nodes are colored by taxa according to the provided key. Clusters are labeled with characterized proteins found within them^17,20,42,44–46,84–87^. **B** The cluster containing ADI, DDAH, AstB, and NnhA proteins in (**A**) shown at an alignment score cutoff >42, showing NnhA as part of a cluster of uncharacterized proteins. **C** The cluster containing NnhA in (**B**) at an alignment score cutoff >50. **D** The cluster containing ADI, AstB, and bifunctional ADI-ornithine cyclodeaminases in **B** shown at an alignment score cut-off >65. **E** Maximum-likelihood phylogenetic tree of representative sequences from the ADI, DDAH, AstB, and NnhA cluster in (**A**) calculated using the LG + G4 model on IQTREE v1.6.12^67^. The clades that represent nodes from the NnhA cluster in (**B**) are highlighted, and the approximate position of branches for the structurally characterized proteins are indicated by their PDB IDs. Nodes with SH-aLRT support values >80 and ultra-fast bootstrap (UFBOOT) values >90 are highlighted with purple circles.

The SSN revealed four distantly related clusters within the GME protein superfamily at a low alignment score cut-off of 25 (Fig. 2A). The first contained the ADI, DDAH, AstB proteins as well as NnhA, indicative of NnhA’s closer evolutionary relationship with these proteins compared to the agmatine deiminase, PAD, and eIF-6 proteins that each form separate and distinct clusters.

Next, a more detailed look at the cluster containing ADI, DDAH, AstB, and NnhA at an increased alignment score cut-off of 42 showed that NnhA clusters with a group of uncharacterized proteins found in bacteria and archaea (Fig. 2B). An alignment score cut-off of 50 for this NnhA cluster revealed that it is composed of multiple smaller clusters (Fig. 2C), and that NnhA is at the tip of a diverging “branch” of proteins predominantly from the phylum Actinomycetota. This suggests that its sequence has diverged substantially from other related proteins.

In addition to NnhA, the SSN also reveals relationships between the other characterized protein families within the GME superfamily. Both prokaryotic and eukaryotic DDAHs form a separate and distinct cluster at the alignment score cut-off of 42 (Fig. 2B), while ADI, AstB, and bifunctional ADI-orthinine cyclodeaminases are all part of the same larger cluster reflecting their higher sequence similarity to each other compared to DDAHs. At an increased alignment score cut-off of 65 (Fig. 2D), this larger cluster of ADI-like proteins separates out into distinct clusters each for ADI and AstB that is reflective of their distinct sequence and functional divergence, while the bi-functional ADI-ornithine cyclodeaminases cluster with and are hence more related to a larger group of uncharacterized proteins. Notably, the ADI proteins are almost exclusively found in bacteria with representation from all common phyla, while AstB proteins are found predominantly in Pseudomonadota.

An unrooted maximum-likelihood phylogenetic tree revealed that the proteins that are most closely related to NnhA form several small clades on the tree near the central node (Fig. 2E), corresponding to the multiple small clusters related to NnhA seen on the SSN (Fig. 2C). Sequences within these small clades and SSN clusters, including the clade containing NnhA, are only represented in a few bacterial and archaeal taxa (Fig. 2C). This suggests that NnhA and closely related proteins are highly specialized and only found in a few biological niches.

In the sequence dataset used for this analysis, the only three closely homologous sequences to NnhA with >50% sequence identity were represented from just the genus *Mycobacteria*. This was investigated further with a BLAST search against the NCBI non-redundant protein database^31^, which revealed only eight sequences with >50% sequence identity to NnhA, six from *Mycobacteria* and two from the other soil bacteria *Amycolatopsis jejuensis and Pseudonocardia spinosispora* (Supplementary Fig. 1). This is despite over 100 mycobacterial species with genomes available in this database, suggesting that NnhA is only rarely found in a few organisms.

The SSN and phylogenetic analysis suggest that NnhA broadly belongs to a group of uncharacterized proteins within the GME superfamily that are distinct from ADIs, DDAHs, and other characterized proteins. However, NnhA appears to have diverged significantly in sequence even among these uncharacterized proteins and is only rarely found in select mycobacteria and other soil bacteria. It is hard to predict without experimental evidence if other distantly related sequences (<50% sequence identity) that cluster with NnhA have 2NI nitrohydrolase activity, which remains to be explored.

### Directed evolution of NnhA for improved expression in *Escherichia coli*

**Fig. 3:**
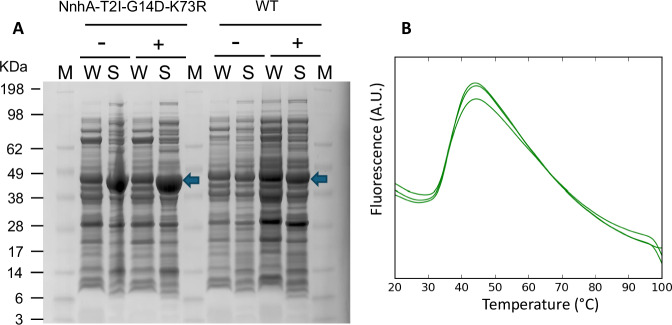
Improving the expression of NnhA. **A** SDS-PAGE gel showing improved soluble expression of the soluble T2I, G14D, K73R NnhA mutant compared to the wild-type (WT) protein before (−) and after (+) induction with Isopropyl β- d-1-thiogalactopyranoside (IPTG). Lanes are labeled M for marker, W for protein from whole cells, and S for soluble proteins. The blue arrow indicates the position of NnhA and its mutant. **B** DSF assay with the variant protein (*n* = 3 independent experiments) showing a T_m_ of 35 °C, in a formulation of 20 mM Tris pH 7, 50 mM NaCl.

To functionally and structurally characterize NnhA, we expressed the protein heterologously with an N-terminal 6x Histidine-tag in *E. coli* strain BL21 λDE3 cells for nickel affinity purification. Our initial attempts at nickel affinity purification of the WT protein led to a heavy white precipitate forming in the collection tubes corresponding to the protein peak, and hence the final protein purification protocol used columns for anion exchange, hydrophobic interaction, and size exclusion chromatography. Regardless, we found that the expression level of soluble protein was very low and not appropriate for crystallization experiments, with at most 0.6 mg of protein produced per liter of *E. coli* culture grown in rich media (Fig. 3A).

We have previously successfully shown that directed protein evolution using phenotypic screening for increased enzyme activity in whole-cell samples can identify variants with improved heterologous expression of soluble protein for triazine chlorohydrolase TrzN^32^. While increased activity in whole-cells can be achieved by an increase in enzyme titer, *k*_cat_ /*K*_M_ value, or both, a low yield of soluble wild-type enzymes, such as in the case of TrzN and NnhA suggests that there would be a relatively large number of amino acid substitutions that would improve the protein titer compared to those that would improve catalytic efficiency. Hence it is likely that such variants would be discovered if a large enough library is screened^32^. Therefore, to identify a variant of NnhA that can be produced in enough quantities in *E. coli* for further functional and structural studies, we performed four cycles of directed evolution on wild-type (WT) NnhA. For this, error-prone PCR was used to generate a library of random *nnhA* mutants (1–3 bp mutations per 1 kbp) that was used to transform *E. coli* BL21 λDE3 cells. NnhA activity was used as a proxy for an increase in NnhA titer, and *E. coli* colonies were screened for improved NnhA activity using a two-step screen. In the first step, bacteria were grown on LB agar plates containing 0.05 mM 2NI, and the largest colonies were selected since more NnhA production with increased 2NI degradation would give a growth advantage. This concentration of 2NI was selected as the minimal inhibitory concentration (MIC) of 2NI for *E. coli* was ~0.1 mM. A sub-MIC concentration elicits a weak to neutral evolutionary pressure, where more NnhA activity and/or expression provides a growth advantage. This is an effective strategy when it is limiting to generate and screen a large enough library to find mutants that can survive at MIC, as demonstrated in our previous work^33,34^. In the second step, whole-cell cultures of the selected colonies were subject to a colorimetric assay using the Greiss reaction^35^, to assess NnhA activity by quantifying nitrite production in the presence of 2NI. Finally, the plasmid DNA from variants with the highest activity was isolated to repeat the mutagenesis and selection process successively three more times, with a higher 2NI concentration of 0.1 mM in the colony selection step to increase the selective pressure. Every round, 22,500 to 40,000 colonies were screened in the first step, with 600 colonies screened in the second step. The five most active variants from the last round were sequenced, and the highest expressing variant was used for further studies (Supplementary Table 1).

Using this process, we identified a variant of NnhA containing three amino acid substitutions (T2I, G14D, and K73R) that exhibited a substantial increase in soluble protein production in *E. coli* compared to the WT when analyzed on an SDS-PAGE gel (Fig. 3A). These substitutions were observed in two of the sequenced variants (SI Table 1). We isolated and purified this variant protein (referred to as “soluble NnhA”) with a yield of 8 mg of protein per liter of *E. coli* culture and compared its catalytic activity with the WT protein (Table 1). This is an improvement of more than a tenfold increase in protein yield compared to the WT protein. The *K*_M_ of the WT and soluble proteins remained similar (93.1 ± 14.1 µM and 109.8 ± 7.6 µM, respectively), suggesting that the substrate binding remains unaffected by the substitutions. However, a 14-fold improvement in *k*_cat_ was observed in the soluble protein (1.27 ± 0.16 min^−1^ and 18.0 ± 0.4 min^−1^ for the WT and soluble proteins, respectively), resulting in a 14-fold increase in *k*_cat_/K_M_ (0.012 µM^−1^ min^−1^ and 0.164 µM^−1^ min^−1^ for the WT and soluble proteins, respectively).

**Table 1:** Enzyme kinetics of 2NI hydrolysis by NnhA and its variants presented in this work, measuring nitrite release using the Greiss reaction as detailed in the methods section

NnhA variant	K_M_ (µM)	*k*_cat_ (min^-1^)	*K*_cat_/K_M_ (µm^-1^ min^-1^)	Notes
WT	93.1 ± 14.1	1.27 ± 0.16	0.012	
T2I-G14D-K73R	109.8 ± 7.6	18.0 ± 0.42	0.164	crystallized
T2I-G14D-K73R-C352A	24.5 ± 3.3	0.73 ± 0.02	0.030	
T2I-G14D-K73R-C352S	n/a	n/a	n/a	Detectable activity, but low titers of soluble enzyme
T2I-G14D-K73R-C357A	n/a	n/a	n/a	no activity, crystallized
T2I-G14D-K73R-C357S	n/a	n/a	n/a	no activity
T2I-G14D-K73R-D262N	n/a	n/a	n/a	no activity
T2I-G14D-K73R-N311D	n/a	n/a	n/a	no activity
T2I-G14D-K73R-H260N	n/a	n/a	n/a	no activity
T2I-G14D-K73R-H260F	n/a	n/a	n/a	no activity

Errors represent standard deviation calculated from *n* = 2 independent experiments.

Despite the increased production of soluble protein, the soluble NnhA variant had only modest stability in solution, with a T_m_ of 35 °C in buffer containing 20 mM Tris pH 7 and 50 mM NaCl (Fig. 3B). Slight increases in stability were seen depending on the buffer conditions during the DSF (SI Fig. 2), mostly marginal up to 40 °C, with piperazine producing the most substantial effect by increasing the T_m_ to 50 °C during the assay. However, the protein precipitated once piperazine was introduced into the enzyme storage buffer and so was not used in downstream experiments.

### Overall structure of NnhA

The purified protein for the soluble NnhA variant crystallized readily in conditions containing polymers, and data were collected using crystals grown in polyacrylic acid or PEG. X-ray data were collected on several crystal forms in different space groups. A combination of single isomorphous replacement with anomalous scattering (SIRAS) using a single mercury derivative and multi-crystal averaging between data in the P1 and H32 space groups were used to solve the structure. With data close to 2 Å resolution (2.04 and 2.16 Å), the quality of the electron density maps was good and showed unambiguous density from residue R11 to R379 at the C-terminus (Table 2). No additional electron density corresponding to metal ions or cofactors was seen, supporting the evidence by enzymatic studies that these are not required for activity^3^. We also crystallized and solved the structure of an active site variant NnhA-T2I-G14D-K73R-C357A, in which the presumptive nucleophilic cysteine residue had been substituted for an alanine residue. Two structures of this variant were solved to a similar resolution in space group H32 using molecular replacement with the model of the soluble NnhA variant (Table 2).

**Table 2:** Data collection and refinement statistics

	NnhA-T2I-G14D-K73R (H32) PDB ID: 9AZG	NnhA-T2I-G14D-K73R (P1) PDB ID: 9AZH	NnhA-T2I-G14D-K73R-C357A (in CHES) PDB ID: 9B01	NnhA-T2I-G14D-K73R-C357A (in Tris) PDB ID: 9B02	NnhA-T2I-G14D-K73R Hg-derivative
**Data collection**					
Space group	H32	P1	H32	H32	H32
Cell dimensions					
*a*, *b*, *c* (Å)	206.4, 206.4, 70.3	59.7, 117.4, 121.1	206.1, 206.1, 68.2	206.1, 206.1, 69.3	206.2, 206.2, 67.5
α, β, γ (°)	90, 90, 120	117.1, 91.3, 101.4	90, 90, 120	90, 90, 120	90, 90, 120
Resolution (Å)	48.7-2.16 (2.23-2.16)	60.7-2.04 (2.07-2.04)	48.0-1.99 (2.04-1.99)	40.3-1.97 (2.01-1.97)	63.1-3.00 (3.18-3.00)
*R*_merge_	0.157 (0.733)	0.222 (0.843)	0.231 (1.011)	0.178 (0.909)	0.329 (0.948)
*I* / σ*I*	28.1 (4.9)	9.6 (2.2)	9.7 (2.7)	10.9 (2.8)	12.1 (5.4)
Completeness (%)	99.9 (99.7)	98.2 (94.6)	99.9 (98.3)	99.8 (96.8)	100 (100)
Redundancy	59.1 (20.2)	7.0 (5.3)	11.3 (10.6)	11.3 (10.6)	33.3 (33.0)
**Refinement**					
Resolution (Å)	48.8-2.16	54.1-2.04	48.0-1.99	40.3-1.97	
No. reflections	29,015	169,055	35,865	37,701	
*R*_work_ / *R*_free_	16.8/20.7	15.6/17.8	14.6/17.4	16.5/19.8	
No. atoms	3136	19,372	3354	3311	
Protein	2931	17,610	2926	2931	
Ligand/ion					
Water	204	1733	414	366	
*B*-factors	29.6	13.9	14.3	18.6	
Protein	31.8	15.0	15.5	20.3	
Ligand/ion					
Water	34.2	21.2	25.6	28.9	
R.m.s. deviations					
Bond lengths (Å)	0.007	0.008	0.008	0.008	
Bond angles (°)	1.553	1.546	1.598	1.438	

^*^One crystal was used for each of the above data sets, and the values in parentheses are for the highest-resolution shell.

**Fig. 4:**
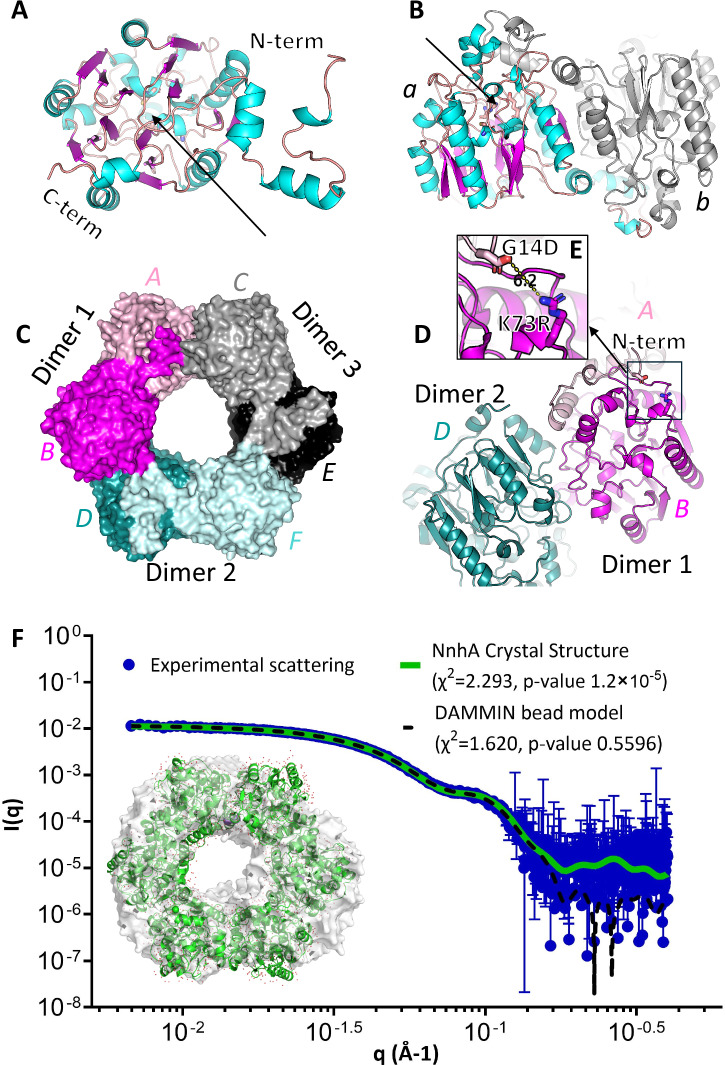
Structure of soluble NnhA (T2I-G14D-K73R) in the P1 space group (PDB ID: 9AZH). **A** Structure of the monomer, showing the overall α/β propeller protein fold (bottom view) colored by secondary structure. The predicted active site residues are shown as sticks, with a black arrow pointing to the substrate binding pocket. **B** Same as (**A**) but side view, showing the “basket with handle” structure. The adjacent protomer in the homodimer is also shown in gray, showing the extended N-terminal α-helix interacting with the “handle” region of the first protomer. **C** The hexameric ring formation, colored by chain and labeled by chain ID in the structure file. Protomers within the same homodimer are shown in light and dark shades of the same color. **D** The three-way interface formed between two homodimers, with dimer 1 (chain A and B) and dimer 2 (chain D and F) labeled. The mutations at the protomer-protomer interface of dimer 1 in NnhA (T2I-G14D-K73R) are also shown as sticks, and the location of the first N-terminal residue that can be resolved (R11) in chain A of dimer 1 (dusty pink) is also shown. **E** Inset from panel (**D**) showing two of the mutated residues G14D and K73R in chains A and B, respectively, with the minimum distance between the functional groups shown as dotted lines and labeled in Å. **F** SEC-SAXS data for soluble NnhA (T2I-G14D-K73R) fitted with the modeled scattering profiles of the crystal structure and the DAMMIN bead model, with an inset showing the surface representation of the DAMMIN bead model in white aligned to the hexameric crystal structure in green. Error bars represent the standard deviation from the mean for *n* = 28 frames of averaged data, and individual data points are provided in Supplementary Data 3.

The protein adopts the α/β propeller fold found in ADIs, DDAHs, and other proteins of the GME superfamily (Fig. 4A), that has a fivefold pseudo-symmetry built with five ββαβ motifs^36^. From the side (Fig. 4B), the loops and α-helix insertions into this core structure gives the appearance of a “basket with handles”^36^. NnhA also has an N-terminal extension that makes extensive interactions with the adjoining protomer to form a domain-swapped homodimer, the presence of which also distorts the first β-sheet in the proceeding ββαβ motif (Fig. 4A, B). The active site of the protein is located toward the top of the core of each protomer where the ββαβ motifs meet, shielded from bulk solvent by a lid formed by a large loop (Fig. 4A, B).

Analysis using PDBe PISA^37^ showed that this protomer-protomer interaction for dimer formation consists exclusively of hydrogen bonding interactions (H-bonds), with a favorable average solvation-free energy gain (ΔG) upon dimer formation of −38.5 Kcal/mol and a high likelihood of being physiologically relevant (Supplementary Table 2 and Supplementary Fig. 3A). Interestingly, two of the three mutations in the soluble NnhA variant, G14D and K73R, are found on the protein surface within this dimer forming interface as well (Fig. 4D, E). The third T2I mutation is not resolved in the structure as it is located at the start of the polypeptide near the N-terminal His-tag. Both G14D and K73R introduce oppositely charged residues that are located such that they could potentially introduce a salt-bridge interaction to strengthen the dimer interface. However, the minimum distance observed between them in all the crystal structures was 6.2 Å, which is further than the expected 2–4 Å for a salt bridge but may still be relevant when the protein is in solution and in a dynamic state. Notably, mutations observed in other variants with improved activity that were sequenced in this study (Supplementary Table 1)—Y74H, K177R, and K231R—are also located in this protomer-protomer interface. Additional mutations observed, V4A and A36T, are located near the N-terminus, with V4A not resolved in the structure and A36T located towards the protein surface. Another mutation, T54A, is located on the hinge between the N-terminal helix and the α/β propeller domain. These observations allude that perhaps stabilization of this dimeric interface contributes to improved soluble expression of NnhA, but this remains to be confirmed by further investigation.

In the unit cell of the P1 space group obtained for soluble NnhA, three homodimers are arranged to create a hexameric ring, with a large pore in the middle (Fig. 4C). There is a single protomer in the H32 space group, but the same domain-swapped hexameric structure is formed when crystallographic symmetry is applied. The two catalytic mutant NnhA C357A structures solved in the H32 space group also adopt the same hexameric structure. This hexamer was also seen in another space group (P21), although these data are not presented here as there was disorder in some regions.

Size exclusion chromatography (SEC) during the protein purification process suggested an approximate molecular weight (MW) of 160 KDa for the protein complex in solution, which suggested the protein may exist as a tetramer based on the 42 KDa MW of the monomer. However, molecular weight calculations with SEC assume a spherical or globular protein complex. This assumption is invalid if the protein exists as a torus/donut-shaped hexamer as suggested by the crystal structure, which would lead to an anomalous molecular weight calculation based on the SEC data. Therefore, we further investigated by performing size exclusion chromatograph coupled small angle x-ray scattering (SEC-SAXS) on soluble NnhA at the Australian Synchrotron BioSAXS beamline.

Based on three concentration-independent methods, the scattering profile indicated that soluble NnhA is hexameric, with the molecular weight estimated to be 242.6 kDa from Bayesian Inference (85.9% probability), 249.24 kDa based on the Volume of Correlation and 259.7 kDa based on the Porod Volume (Supplementary Table 3). When validating the hexameric crystal structure against the SEC-SAXS profile, the modeled scattering profile of the crystal structure had a *χ*^2^ value of fit to the experimental SEC-SAXS profile of 2.264 but a corMAP *p* value of 8.75 × 10^−11^ (Fig. 4F). This suggests that the crystal structure is not a perfect representation of the SEC-SAXS data according to the statistical cut-off values that are commonly accepted^38^, where χ^2^ < 2 and corMAP *p* value >0.02 indicates a good fit of the calculated scattering profile of a model to the experimental SEC-SAXS profile. A discrepancy like this is not unusual when comparing SEC-SAXS data with crystal structures and most likely arises from conformational differences between proteins in solution compared to when crystallized^39^. The conformational flexibility within the oligomerized complex was also supported by the crystal structure, where high B-factors were observed in surface residues of some protomers but not others (Supplementary Fig. 5). For further validation of the hexameric complex in solution, we used the SEC-SAXS data to generate an ab-initio low-resolution DAMMIN bead model of the molecular envelope of the protein, which was generated such that comparison of the calculated scattering profile of the final DAMMIN model and the experimental SEC-SAXS data fell within the accepted statistical cut-off values (*χ*^2^ value of 1.620 and a corMAP *p* value of 0.5596). The resulting torus/donut-shaped structural manifold of the DAMMIN model closely resembled the hexameric crystal structure (Fig. 4F), which along with the confidence of the MW estimates supports the hexameric nature of NnhA in solution like in the crystal structures.

There are three dimers found in the hexameric structure, and therefore each protomer has interactions with three other protomers, including its dimerization partner (Fig. 4C, D and Supplementary Fig. 3). Apart from the dimer forming interaction (Supplementary Fig. 3A), the main interaction between dimers is the interaction between the α/β propeller core of two adjacent protomers that form two different dimers (Supplementary Table 2 and Supplementary Fig. 3B). This interaction is formed by both H-bonds and salt bridges, and although calculated to be physiologically relevant by PDBe PISA, it had a lower and comparatively less favorable average ΔG upon interface formation (−6.9 Kcal/mol) than the dimer formation interaction between protomers (−38.5 Kcal/mol) (Supplementary Table 2). The second minor interaction at the dimer-dimer interface is between the N-terminal extension of one protomer and the α/β propeller core of the protomer adjacent to its dimer partner, in other words, the protomer once removed (Supplementary Fig. 3C). With an average ΔG of interface formation at just −2.1 Kcal/mol, PDBe PISA calculates the significance of this interaction to be borderline (Supplementary Table 2).

### Structural comparison of NnhA to ADI and DDAH

**Fig. 5:**
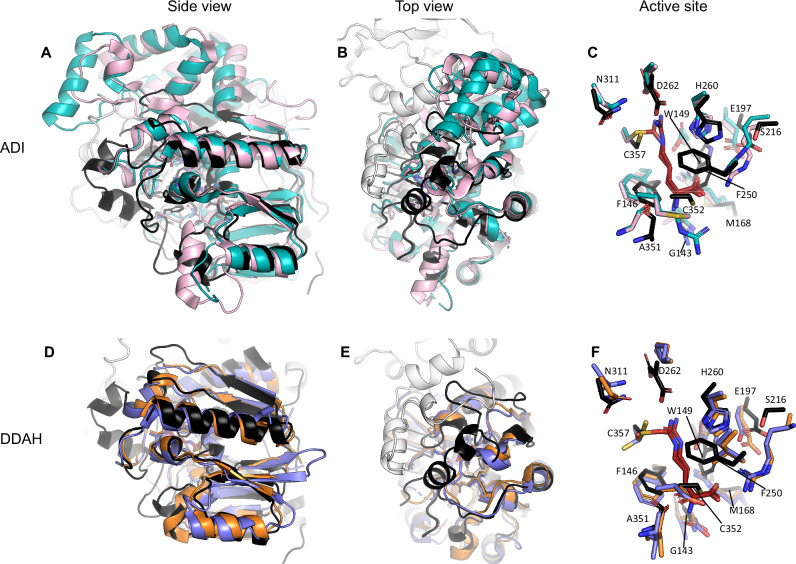
Comparison of the structure of NnhA to characterized ADI and DDAH proteins. **A**–**C** shows alignment of NnhA (black) to ADI from *Pseudomonas aeruginosa* (teal, inactive mutant C406A, PDB ID: 2A9G^43^) bound to arginine (red), and to ADI from *Mycoplasmopsis arginini* (pink, PDB ID: 1S9R^20^) covalently bound to the *S*-alkythiouronium reaction intermediate of arginine (red). **D**–**F** shows NnhA (black) aligned with the C249S mutant of DDAH from *Pseudomonas aeruginosa PAO1* (orange, PDB ID: 1H70^17^) bound to citrulline (red)^17^, and with human DDAH (blue, PDB ID: 2JAI^42^) covalently bound to citrulline (red). For clarity, only the residues from NnhA are labeled, where the catalytic residues are C357, H260, and E197.

Comparison of the protomer of the soluble NnhA structure to existing structures in the protein databank using FoldSeek^40^ produced 100 hits with a probability score of 1.00, where the closest related structures to NnhA were of ADIs and DDAHs, but with only low sequence identities of ~12–17%. The overall structural alignment was also poor across all the structures with ~2–3 Å root mean square deviation (RMSD) over about 200 Cα atoms that mostly resided in the α/β propeller core (Fig. 5A,  D). There was no alignment found for the dimer forming N-terminus. Indeed, neither DDAHs nor ADI proteins are known to form a domain-swapped dimer like NnhA. The most closely related structure was that of the C249S mutant of DDAH from *Pseudomonas aeruginosa PAO1* bound to citrulline (PDB ID: 1H70^17^) with 16.8% sequence identity, an RMSD of 2.11 Å over the Cα atoms of the 253 aligned amino acids, and a TM-score^41^ of 0.89.

From the related structures found by FoldSeek^40^, we picked structures of DDAH and ADI co-crystallized with the substrate or an *S*-alkythiouronium reaction intermediate to compare and identify the substrate binding pocket of NnhA. In addition to the *P. aeruginosa* DDAH structure (PDB ID: 1H70^17^), these were the structures of human DDAH covalently bound to citrulline (PDB ID: 2JAI^42^), the inactive C406A mutant of ADI from *P. aeruginosa* bound to L-arginine (PDB ID: 2A9G^43^), and ADI from *Mycoplasmopsis arginini* covalently bound to the *S*-alkythiouronium reaction intermediate of arginine (PDB ID: 1S9R^20^).

The differences between NnhA and these ADI and DDAH proteins highlight the evolutionary divergence of NnhA from these proteins (Fig. 5). At first glance, despite an overall similar core structure, it is apparent that NnhA contains two additional α-helices forming the lid of the α/β propeller core that covers the active site opening that are absent from the ADI and DDAH (Fig. 5A, B, D,E). Additionally, one of the two domain-swapped helices from the N-terminus of the dimerized protomer is also found near this region, making the active site of NnhA less exposed to bulk solvent than the ADI and DDAH active sites.

Comparing the NnhA protomer with both the *P. aeruginosa* and *M. arginini* ADIs shows that the core α/β propeller domains of both ADIs align with that of NnhA (2.92 and 3.03 Å, respectively) (Fig. 5A, B). However, the ADIs contain an α-helical domain adjacent to the core α/β propeller domain^20,43–46^, which is absent in NnhA. Comparison to both the *P. aeruginosa PAO1* and human DDAH structure also reveals that NnhA has a good overall alignment in the core α/β propeller domain (2.11 and 2.53 Å, respectively), with no additional domains present in the DDAH structures (Fig. 5D, E).

The residues in the active site of NnhA appear somewhat conserved in comparison to the ADI and DDAH, with the catalytic histidine, glutamic acid, and cysteine residues present in NnhA as H260, E197, and C357 (Fig. 5C, F). Interestingly, a second cysteine residue (C352) is also present in the substrate binding pocket of NnhA (Fig. 5C, F). This residue is not conserved in the other proteins and is replaced with arginine and methionine residues in the *P. aeruginosa* and *M. arginini* ADIs, respectively, and to aspartic acid in both the *P. aeruginosa PAO1* and human DDAHs.

To investigate which of these cysteine residues is likely to be the catalytic nucleophile for the reaction, we substituted either C352 or C357 for either alanine or serine residues in addition to the T2I, G14D, and K73R mutations (Table 1). We found that despite soluble expression, C357A and C357S substitutions eliminated activity. On the other hand, the C352A variant still retained activity with fivefold lower catalytic efficiency than the base NnhA-T2I-G14D-K73R variant, and the C352S variant also retained detectable activity despite its poor soluble expression that prevented protein purification for detailed enzyme kinetics. This suggests that of these two cysteine residues, C357 is more likely to be the nucleophile in NnhA, fulfilling a similar role to the nucleophilic cysteine in ADI and DDAH that are conserved in the same structural position (Fig. 5C, F)^23,24^. We also mutated H260, which is the equivalent residue to the catalytic base in ADI and DDAH, to either asparagine or phenylalanine, and found that both mutations lead to inactive protein variants, suggesting a similar role in NnhA.

Comparing the other residues at the active sites, D262 of NnhA is conserved in both ADIs, where the equivalent residues in the ADIs stabilize the guanidino moiety of the substrate (Fig. 5C). In both DDAHs, this is a lysine residue (Fig. 5F), which allows accommodation of the dimethyl amino-group of the DDAH substrate^42^. N311 in NnhA is also conserved in the ADIs and DDAHs (Fig. 5C, F), where it provides stability to other active site residues^24^. A second aspartic acid residue stabilizes the guanidino moiety from the opposite side in ADI and DDAH, although this residue is W149 in NnhA instead (Fig. 5C, F).

NnhA also lacks the residues in the compared ADI and DDAH structures that form polar interactions with the carboxylic and amine group of arginine and arginine-derived substrates. S216 and M168 in NnhA replace the arginine residues of the ADIs and DDAHs that stabilize the carboxyl moiety of the substrate, and G143 in NnhA replaces aspartic acid in the same position in the ADIs and DDAHs that interact with the amine group of the substrate (Fig. 5C,  F). The M168, G143, and W149 substitutions in NnhA, along with residues like F146 and F250, create a hydrophobic substrate binding pocket, which could facilitate the binding of the aromatic imidazole moiety of 2NI.

### Reaction mechanism of NnhA compared to other GME superfamily enzymes

To better understand the reaction mechanism of 2NI hydrolysis by NnhA, we tried to co-crystallize and soak 2NI into both the active and inactive (C357A) soluble NnhA crystals. However, no excess density for the 2NI moiety could be found in any of these structures. The only notable difference between the apo structures of the two variants was in the conformation of D262 at the active site (Supplementary Fig. 6).

While D262 exists in two rotomeric conformations in soluble NnhA, only one conformation with a slight displacement is seen in the structure of the C357A mutant of soluble NnhA. Hence, it is possible that D262 has mobility within the active site, which may play a role in the catalytic mechanism.

For further insight into the mechanism and roles of the active site residues, a 2NI molecule was docked into a protomer in the structure of the homodimer of the catalytically active soluble NnhA variant using AutodockFR^47^. However, only very low free energy for binding (−3.7 kcal/mol) was achieved for any plausible binding mode with C357 within a reactive distance (~3–4 Å) from C2 of 2NI (Supplementary Fig. 6), suggesting a conformational change maybe required in the active site for successful binding.

**Fig. 6:**
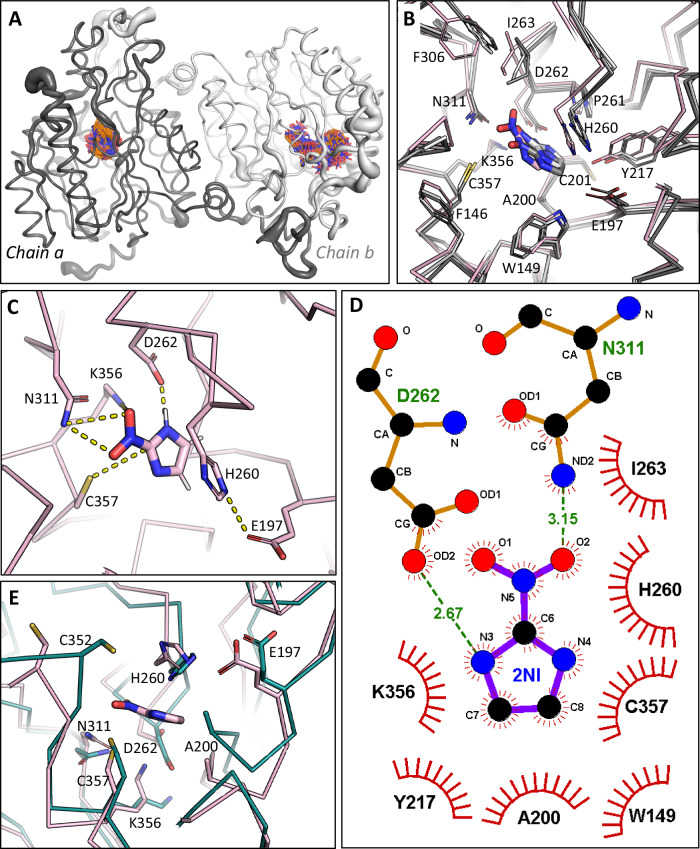
Substrate binding in NnhA. **A** Mobility of the 2NI bound soluble NnhA homodimer during the 500 ns MD simulation. The protein backbone is represented by noodles, the thickness of which represents higher B-factors in more mobile residues. The 2NI molecules bound to each protomer are shown as sticks, representing their position at every 1 ns of the simulation. **B** An overlay of the representative (gray), averaged (white), and minimal C357 to 2NI (C2) distance (pink) structures from the second most populated cluster (13.0%) from cluster analysis of the MD trajectory. The binding site shown is for chain A. **C** Catalytic residue interactions with 2NI in the minimal distance structure shown in panel (**B**). **D** Protein-ligand interaction map generated by LigPlot+^88^, showing the residues that interact with 2NI in the binding pose shown in (**C**). Hydrogen bonding interactions are shown in green with atomic distances provided in Å, and all other interacting residues are also stated. **E** Minimal distance structure in panel (**B**) overlayed with chain A of the crystal structure model of soluble NnhA (dark teal) to highlight conformational changes in the substrate binding site to accommodate 2NI.

Hence, we performed a 500 ns molecular dynamics simulation (Amber20) of the soluble NnhA homodimer docked with 2NI in each protomer. The system showed equilibration between 50 and 500 ns for three independent simulation runs, with convergence seen when comparing both backbone RMSD over time and per residue B-factors (Supplementary Fig. 7A,B). Consistent with the low free energy of binding predicted from the molecular docking, both 2NI molecules had a high degree of mobility during the simulations (Fig. 6A), with average B-factors of 117 ± 52 and 312 ± 199 Å^2^ for 2NI in chain A and chain B, respectively, compared to the average of 41 Å^2^ observed in the protein backbone atoms (Supplementary Fig. 7B). The protein backbone demonstrated highest mobility in residues that could allow substrate access from the protein surface such as residues 85–102, 240–260, and 340–355, which was more pronounced in chain B where the 2NI molecule was less stabilized and sampled more conformations closer to the protein surface (Fig. 6A). Similar regions of the protein (residues 70–120 and 330–360) were also observed to have higher B-factors in the hexameric crystal structure but only in some protomers, supporting that these regions have high conformational flexibility that is likely to be important for substrate binding.

To identify a plausible 2NI binding mode that would facilitate the hydrolysis reaction, we performed cluster analysis on the subset of atoms comprising 2NI along with C357, N311, and A200 in the substrate binding site of chain A. Clusters were obtained until the minimum distance between clusters was greater than 1.0 Å. A total of 66 clusters were identified, although 90% of frames were classified into the first 15 clusters (Supplementary Fig. 7C and Supplementary Table 4). The most populous cluster contained 23.1% of frames, but the shortest distance observed between C357 and C2 of 2NI was 4.76 Å, which is further than the 3–4 Å for Van der Waal’s contact in a near attack conformation^48^. Analysis of the binding pose in the second most populous cluster containing 13.0% of frames suggested potential reaction facilitation, with the minimum distance between C357 and C2 of 2NI at 3.77 Å (Fig. 6B, C). In this structure, the amide group of N311 stabilizes the nitro moiety of 2NI along with K356, and the protonated nitrogen atom on the imidazole moiety is stabilized by D262 (Fig. 6C and Supplementary Fig. 8). These interactions are accommodated by conformational changes in the binding site compared to the crystal structure, particularly in the backbone atoms of residues D262, A200, and C352 (Fig. 6D). To investigate the role of N311 and D262 in substrate interaction, we expressed and purified the variants of soluble NnhA D262N and N311D. Both variants did not exhibit catalytic activity despite soluble expression (Table 1), consistent with a role in substrate binding and/or reaction catalysis. Notably, the substrate binding pose in the first larger cluster did not involve interactions with these residues (Supplementary Fig. 8).

The catalytic H260 and E197 residues required for the hydrolysis step of the reaction are positioned on the opposite side of the 2NI molecule to the nucleophile C357 (Fig. 6B, C). More precisely, E197 is positioned towards C4 and C5 on the imidazole ring of 2NI, which may explain why NnhA appears specific towards 2-nitroimidazoles such as 2NI and benznidazole^3^. It is however not active with 4-nitroimidazoles, 5-nitroimidazoles, 2,4-nitroimidazoles, or thiazolidines^3^, which could be because binding the nitro moiety of 4- and 5-nitroimidazoles would sterically hinder the positioning of E197. The rest of the substrate binding pocket contains mostly hydrophobic residues (Fig. 6B and Supplementary Fig. 8), notably F146, W149, C201, Y217, P261, I263, and F306, that are likely to stabilize 2NI through Van der Waal’s interactions.

**Fig. 7:**
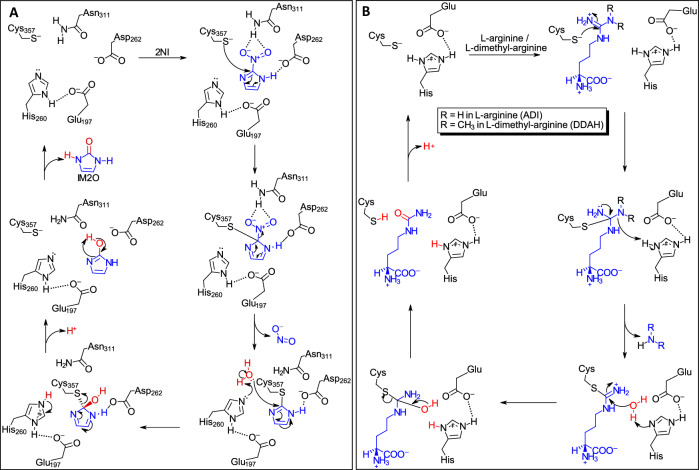
Proposed reaction mechanism for NnhA compared to ADIs and DDAHs. **A** Proposed reaction mechanism for 2NI hydrolysis by NnhA. **B** General reaction mechanism of ADIs and DDAHs as previously described in refs. ^17,18,20,44,49^.

Based on the observations from the crystal structures, MD simulation, and previous studies on ADI^18,20,44^ and DDAH^17,49^, and other GME superfamily enzymes^23,24^, we propose that NnhA catalyzes 2NI hydrolysis via a similar reaction mechanism **(**Fig. 7A) as ADI and DDAH (Fig. 7B). In both ADI and DDAH (Fig. 7B), a first tetrahedral intermediate forms upon nucleophilic attack by the catalytic cysteine, followed by elimination of ammonia or mono-/di-methylamine in ADI or DDAH, respectively, to form a covalent intermediate. This is followed by the formation of a second tetrahedral intermediate through nucleophilic attack by a water molecule activated by the stabilized histidine-glutamic acid base, which leads to substrate release upon elimination of the cysteine residue.

Similarly, in NnhA (Fig. 7A), nucleophilic attack from C357 on C2 of 2NI could form a covalent tetrahedral intermediate, stabilized by hydrogen bonding by D262. Next, NO_2_^-^ leaves the complex so that the imidazole ring of the substrate forms a planar covalent intermediate with C357. This can then allow nucleophilic attack by a water molecule that is activated by the catalytic base E197-H260 on C2 of the planar covalent intermediate. We modeled this covalent intermediate using covalent docking of imidazole to C357 in chain A of the NnhA-T2I-G14D-K73R homodimer using AutoDockFR^47^. The crystal structures from both space groups show that at least one protomer in every structure obtained in this study contained electron density for a water molecule within hydrogen bonding distance (2–3 Å) of H260, that would be also within 2–3 Å of C2 of the covalent intermediate (Supplementary Fig. 9). Thus, the second tetrahedral covalent intermediate can be formed, aided by hydrogen bonding by D262. C357 would be the leaving group this time, breaking the covalent bond between the substrate and enzyme to make the *enol* form of IM2O, which can readily tautomerize to the *keto* form to make the final product. Further substrate binding simulations and mechanistic studies are required to confirm this proposed reaction scheme.

This mechanism is reminiscent to that of cysteine proteases that contain a cysteine-histidine dyad where the cysteine residue is first deprotonated by histidine, prompting nucleophilic attack on the amide bond of the substrate to form a tetrahedral intermediate^50,51^. This then breaks the amide bond to release the amine terminus of the substrate while leaving behind a covalent thioester intermediate, which is hydrolyzed by a water molecule via a second tetrahedral intermediate to release the carboxylic acid product. However, unlike cysteine proteases but similar to other GME superfamily enzymes, the catalytic cysteine of NnhA is located ~5 Å from the histidine-glutamic acid base, which is hence unlikely to be able to deprotonate the cysteine to initiate the reaction. In DDAH from *P. aeruginosa*, the catalytic cysteine is thought to be deprotonated to the ionic thiolate by the positively charged guanidium of the substrate in a substrate-assisted nucleophile activation mechanism^49^, but the presence of similar mechanisms in other GME superfamily enzymes such as NnhA remains to be investigated.

To summarize, NnhA is found in a small, largely uncharacterized cluster of homologs within a large family of enzymes with deaminase and related activities. It displays an unconventional hydrolytic mechanism for denitration, potentially useful in biodegradation and bioremediation, including dealing with nitroaromatic explosives with related structures like 2,4-dinitroimidazole, 4,4′,5,5′-tetranitro-2,2′-bisimidazole, and 2,4,5-trinitroimidazole. The structural and mechanistic insights presented here will facilitate the development of such technologies.

The nitroimidazole antibiotics are unusual in that two separate mechanisms for resistance have been identified before being observed in a clinical setting. Nitrohydrolase activity by NnhA confers metabolic resistance to these drugs^3^, and mutations in nitroreductases such as Deazaflavin dependent nitroreductase (Ddn) produced by *Mycobacterium tuberculosis* prevent prodrug activation^48^. The active moieties in most (if not all) antibiotics are based on natural antimicrobial compounds, and it is unsurprising that resistance to such natural antimicrobials evolved long before the antibiotic revolution of the mid-twentieth century. This reservoir of genetic resources allows the rapid evolution of resistance to semisynthetic antibiotics via the strong selection pressure conferred by their use. Understanding resistance mechanisms prior to large-scale deployment of an antibiotic class could lead to preemptive strategies to manage antibiotic use and slow resistance development.

## Experimental methods

### Protein expression and purification

The *nnhA* gene was codon optimized for expression in *E. coli* and synthesized by GeneArt (Invitrogen). The coding region of NnhA was released from the cloning vector pMA-T (Invitrogen) by digesting with Nde I and Eco RI and ligated into the same restriction sites of expression vector pETMCSIII^52^. The vector pETMCSIII allows leaky expression in *E. coli* host strain BL21 (DE3) (New England Biolabs). The pETMCSIII with an insert of the NnhA coding sequence was used for all aspects of this work, including the production of NnhA and mutagenesis.

Transformants of the WT and mutant constructs were incubated at 28 °C overnight in LB medium containing 100 mg/mL of ampicillin and 1 mM of lactose. Cells were harvested and resuspended in 20 mM Tris pH 7.0. The cells were lysed using a homogenizer (Microfluidics), and insoluble materials were removed by centrifugation (Beckman Coulter) at 39,190×*g* at 4 °C for 45 min. Purification was carried out with an ÄKTA chromatography system from GE Healthcare. Initially, we attempted purification of the WT protein with nickel affinity chromatography but found that it led to a heavy white precipitate forming in the tubes corresponding to the protein peak. Hence subsequent purifications of all protein variants did not use a nickel affinity column. Instead, the clarified crude lysate was loaded onto a Q-Sepharose HP column (75 mL) and eluted with a gradient of 0–100% of 1 M NaCl buffered with 20 mM Tris pH 7.0 in 5x column volumes. Fractions were collected and protein contents were inspected by SDS-PAGE. The fractions containing the NnhA protein were pooled and mixed with the same volume of 3 M ammonium sulfate before loaded onto a SOURCE 15 PHE column (15 mL) and eluted with 100%–0 gradient of 1 M ammonium sulfate buffered with 20 mM phosphate at pH 7.0 in 10× column volumes. Fractions containing NnhA were combined and loaded onto a Superdex 200 prep column (120 mL) and eluted with 1.2× column volume of 20 mM Tris pH 7.5 and 50 mM NaCl. The NnhA protein was concentrated using an Amicon Ultra-15 (Merck). Protein was concentrated to 14 mg/mL in 20 mM Tris pH 7, 50 mM NaCl and was snap frozen in 50 µL aliquots and stored at −80 °C until use.

### Library creation

Error-prone PCR (ep-PCR) used to introduce random mutations has been described elsewhere^53^. The ep-PCR reaction was consisted of 1 µM of forward primer pET3 (5′-CGACTCACTATAGGGAGACCACAAC-3′) and reverse primer pET4 (5′-CCTTTCGGGCTTTGTTAGCAG-3′), 50 ng of plasmid, 1x Taq DNA polymerase buffer (Bioline), 5 mM MgCl_2_, 0.1–0.4 mM MnCl_2_, 0.5 mM dNTPs (Bioline), 5 U Taq DNA polymerase (Biloine), and Milli-Q water to final volume of 50 µL. Ep-PCR was performed with conditions of 50 cycles (94 °C for 10 s, 45 °C for 10 s, and 72 °C for 20 s). The ep-PCR product was digested with NdeI and EcoRI and ligated into the same restriction sites of pETMCSIII. The plasmid DNA of pETMCSIII containing mutated *nnhA* gene was transformed into BL21(DE3) by electroporation using Gene Pulser (Bio-Rad). The final library used for screening contained 1–3 bp mutations per 1 kbp.

### Library screening and NnhA activity assay

The primary screen was devised with selection by 2NI which is a natural antibiotic. The library of randomly mutated NnhA was plated out with 300–400 colonies on an LB agar plate containing 100 mg/mL of ampicillin. Typically, 75–100 plates were used for each round of directed evolution. The concentration of 2NI used for selection was 0.05 mM for the first round, which was then increased to 0.1 mM for the following rounds of evolution. After incubation at 30 °C for 72 h, 4–5 larger colonies were picked from each plate for secondary screening.

The secondary screen was performed using a colorimetric assay of nitrite with Griess Reagent System (Promega) in a 96-well format. The colonies picked from selection plates in the primary screen were inoculated in 300 µL of LB containing 100 mg/mL of ampicillin and 1 mM of lactose and incubated overnight at 30 °C. About 50 µL of cell culture was mixed with 50 µL of 0.4 mM 2NI. After incubation at 25 °C for 30 min, 100 µL of 1% sulfanilamide in 5% phosphoric acid was added. After another incubation at 25 °C for 15 min, 100 µL of 0.1% *N*-1-napthylethylendiamine dihydrochloride was added, and absorbance at 540 nm was measured using a microtiter plate reader (Spectramax M2e). 28–44 of the most active variants from the secondary screen were selected, and plasmid DNA was isolated for the next round of evolution. After four rounds of evolution were performed, the plasmid DNA of the best variants was isolated and sequenced (Macrogen).

### Enzyme activity assays

The kinetics of hydrolysis of 2NI by the purified WT, T2I-G14D-K73R, and T2I-G14D-K73R-C352A NnhA variants were also obtained by the Greiss reaction as described above. Reactions were performed for 2NI concentrations of 25, 50, 100, 200, and 400 μM, in the presence of 1.1 μM of WT or NnhA-T2I-G14D-K73R or 3.3 μM of NnhA-T2I-G14D-K73R-C352A. Absolute initial reaction velocities (µM min^−1^) were calculated using a reference curve with the nitrite standard prepared according to the manufacturer's instructions (Promega). The kinetic constants were determined using Michaelis–Menten plots of the data collected.

### Site-directed mutagenesis

Site-directed mutagenesis was performed using the Q5 Site-Directed Mutagenesis Kit (NEB). Mutations were confirmed by DNA sequencing (Macrogen).

### Differential scanning fluorimetry (DSF)

Soluble NnhA protein was used to set up thermal melt experiments to test the pH sensitivity and stability of the protein construct. 300 µL of protein at 2 mg/mL in 20 mM Tris pH 7, 50 mM NaCl, along with 300 µL of 20× diluted Sypro Orange dye (Sigma S5692) were diluted into a final volume of 20 µL in 96-well plates (Thermo AB0800W). Triplicate trials were run testing different buffer/pH and salt concentrations by heating in a standard RT-OCR machine (Bio-Rad CFX96), following a previously published protocol^54^. Results were interpreted using the Meltdown visualization package^55^.

### Protein crystallization

The proteins (both active and inactive mutants) precipitated upon thawing on ice but came back into solution when diluted from 14 mg/mL 1:4 into 20 mM Tris pH 7, 50 mM NaCl. The reconstituted protein at 3.5 mg/mL was set up in sitting droplets (200 nL protein, 100 nL reservoir) in SD-2 plates (Molecular Dimensions, UK) using the Shotgun screen at both 20 and 8 °C, and crystallized in several different polyethylene glycol containing conditions overnight. Further aliquots of frozen protein were diluted 1:2 in either 20 mM Tris pH 7, 50 mM NaCl or 50 mM CHES pH 9, 50 mM NaCl, and these two samples were set up in sitting drops (300 nL protein, 150 nL reservoir) against four 96-well screens, which were incubated at 20 °C. Leftover protein was set up in 1 μL + 1 μL hanging droplets against 24 conditions from the JCSG screen and incubated at room temperature. Although the protein crystallized readily, most crystals were poorly formed. Large, diffraction-quality crystals were obtained when the protein at 7 mg/ml was added to 21–22% w/v polyacrylic acid 5100, 20 mM MgCl_2_, and 100 mM HEPES pH 7.2. Further rod-like crystals were also obtained from a condition containing 20% PEG 3350 with 240 mM sodium malonate-malonic acid pH 7.0. The C157A mutant protein was diluted to 2.5 mg/mL in either Tris or CHES buffer and then set up in crystallization trials with polyacrylic acid 5100 at 20 °C in 200 nL plus 200 nL drops. The Tris-buffered protein crystallized in 15 mM MgCl_2_, 21.7% polyacrylic acid 5100, and 100 mM HEPES pH 7.2. The CHES buffered protein crystallized in 30 mM MgCl2, 21.2% polyacrylic acid 5100, and 100 mM HEPES pH 7.2. Of the many cryoprotection agents trialed, diethylene glycol gave the best diffraction.

### Protein structure determination

All data sets were collected from a single crystal at 100 K, and both the MX1 and MX2 beamlines of the Australian Synchrotron were used for data collection. The Hg-derivative was collected at wavelength 1.0080 Å, the native H32 crystal used for SIRAS with this data set was collected at 0.9184 Å, the higher resolution H32 crystal was collected at 1.4586 Å, the P1 crystal was collected at 1.1816 Å, and the two mutant crystal structures were collected at 0.9537 Å. There were no Ramachandran outliers for any of the structures, and the Ramachandran plots showed that 96–98% of all residues for a given structure were in the favored regions.

Multiple crystals were harvested and yielded data sets with varying diffraction limits and space group symmetries. The first crystals to show diffraction were indexed in C222_1_ and diffracted to ~3 Å but had a very long cell axis (60 × 218 × 598 Å). Subsequently, crystals grown under different conditions diffracted to higher resolution and had smaller cells. The H32 crystals were reproducible, had a cell of 206 × 206 × 70 Å, and showed diffraction spots beyond 2 Å. The P1 crystals have a cell of 59.7 × 117.4 × 121.1 Å with angles of 117.1 × 91.3 × 101.4 (Table 2).

The initial structure was solved using AutoRickshaw^56^ by SIRAS, with data sets from soluble NnhA crystals, one soaked with HgCN and both in the H32 space group. The data went to 3.0 Å for the HgCN-soaked crystal (2 × 360 degrees of data, so had high multiplicity), and the soluble NnhA crystal also had 360 degrees of data to 2.28 Å. The partial model output by AutoRickshaw was then used for molecular replacement (MR) with Phaser^57^, to a high-resolution (2.04 Å) data set of soluble NnhA in a P1 space group with 6 molecules in the asymmetric unit. The density was significantly stronger (multi-crystal and NCS averaging), and this structure was partially rebuilt and refined. The subsequent model was then used again as an MR model (with Phaser) into a higher resolution H32 space group data set at 2.16 Å with only one molecule in the asymmetric unit.

All data were processed with XDS^58^, all manual model building was done using the program COOT^59^, and all refinement was done with REFMAC^60^. The models were checked using validation tools in Coot and using the validation tools during deposition into the RCSB. Structures and structure factors are available in the PDB with accession codes: 9AZG, 9AZH, 9B01, and 9B02.

### Sequence similarity networks and phylogenetic analysis

The sequence similarity network (SSN) was made using the Enzyme Function Initiative Sequence Similarity Tool (EFI-EST)^27^, by providing the sequence of NnhA (Uniprot ID: F4ZCI3.1) and the sequences for proteins from the GME protein superfamily (Pfam ID: CL0197). Only sequences longer than 200 amino acids were included, and the initial alignment score cut-off used was 25. The network with each node representing proteins with >45% sequence similarity was visualized using Cytoscape 3.9.0^61^, with the yFiles Organic layout. The alignment score cut-off visualized was incrementally changed, followed by network visualization to identify protein clusters likely to represent functional groups.

To make the phylogenetic tree, sequences for one representative protein from each node from the large cluster containing NnhA, DDAH, ADI, and AstB at an alignment score cutoff >25) was retrieved from NCBI and uniprot protein databases. CD-Hit^62^ was used to remove sequences with >90% sequence identity, and any sequences of special interest like NnhA and solved PDB structures were added, to make a set of 4322 sequences. Multiple sequence alignment (MSA) of the sequences were visualized using Unipro UGENE v. 43.0^63^. Sequences were initially aligned using MAFFT^64^, and any partial sequences and those with long inserts were removed. This was followed by iterative alignment with MAFFT and removal of columns with large gaps, until only columns with <10% of gaps were left. Any sequences missing the conserved catalytic cysteine residue were also removed. Approximate maximum-likelihood phylogenetic trees were constructed from the resulting MSA using the LG model on FastTree2.0^65^, where any obvious long branches were removed, and the process was repeated until the tree topology remained stable. The tree was then trimmed with TREEMMER v0.3^66^ to 0.5 RTL, which gave a final sequence set of 804 sequences, that was input to IQTREE v1.6.12^67^ to produce a tree using the calculated best model (LG + G4). A final iteration of the sequence list to 799 sequences based on this initial tree removed any long branches. The presented tree was calculated on IQTREE v1.6.12 with the LG + G4 evolutionary model, with UFBOOT (1000 alignments) and sh-like aLRT (1000 replicates) node support values.

### Small angle X-ray scattering

The SAXS experiments for this work were carried out on the BioSAXS beamline at the Australian Synchrotron ANSTO. Samples were measured using the Coflow sample autoloader in size exclusion chromatography (SEC) mode^68^. The Coflow autoloader utilizes the principles of coaxial flow of the sample in a sheath fluid to mitigate radiation damage to the samples^69^.

Prior to exposure to the X-ray beam, 50 µL of purified soluble NnhA at 3.50 mg/mL was loaded onto a Superdex S200 5/150 GL column (Cytiva) and an Azura Pump P 6.1 L quaternary pump (Knauer, Berlin, Germany) was used to perform size exclusion chromatography at a flow rate of 0.4 mL/min. The eluent from the column was passed through a 3 mm UV flow cell (Knauer, Berlin, Germany) for detection of UV absorbance at 260 and 280 nm by an STS microspectrometer (Ocean Optics, Orlando, FL, USA) immediately before being presented to the X-ray beam. A 12.4 keV X-ray beam energy (λ = 1.00 Å) was used, along with a Pilatus3 X 2 M detector. The sample-to-detector distance was 3151 mm, covering a q-range of 0.007–0.475 Å^−1^. Data were reduced by Fast Azimuthal Integration using Python (PyFAI) with customized algorithms written for the BioSAXS beamline. Data were placed on an absolute scale using water in the measurement capillary as a standard and the nominal diameter of the capillary at the measurement position was 1.0 mm.

All data processing was performed in BioXTAS-RAW version 2.3.0^70^ with ATSAS 3.2.1^71^. The SEC scattering profile was buffer subtracted and baselined in BioXTAS-RAW^72^, and the P(r) function was calculated using GNOM^73^ with AutoRG^74^. Concentration-independent molecular weight estimates were calculated using Bayesian Inference^75^, Volume of Correlation^76^, and corrected Porod volume^77^. Assessment of the crystal structure to the scattering profile was performed using CRYSOL^78^, and dummy atom models were created in DAMMIN^79^ with averaging in DAMAVER and refinement in DAMFILT^80^. SAXS sample details, data collection, analysis, and 3D modeling details are provided in Supplementary Table 3, while SAXS profiles are provided in Supplementary Fig. 4.

### Molecular modeling

Chain A and Chain B from the structure of soluble NnhA in the P1 space group was used for the molecular docking and MD simulations, with the missing 18 residues from the N-terminus left unmodelled in both chains. The 3D structure of 2NI was obtained from PubChem^81^ and docked into the active site pocket of chain A using the standard protocol on AutodockFR^47^, with flexible side chains for the residues S148, W149, E197, H260, F262, and C357. For the docking, the receptor and ligand were prepared using the standard protocols in AutoDockTools^82^, and the affinity maps were calculated using the graphical user interface of AutoGridFR^47^. The docked 2NI was then superposed to chain B to make the docked homodimer a starting point for the MD simulations. In both chains, C352 and C357 were deprotonated, and H260 was only protonated at the ε-nitrogen.

Molecular dynamics simulations of 500 ns were carried out using the AMBER20 package^83^. The Generalized Amber Forcefield (GAFF2) was employed for the 2NI molecules, and the all-atom FF14SB forcefield was used for the protein, which, in combination with the TIP3P solvation model, is widely accepted to accurately predict protein-ligand binding interactions^84^. The structure was solvated in a periodic octahedral TIP3P water box with a minimum solute-boundary distance of 12 Å, and charge neutralized with Na^+^ ions (from frcmod.ionsjc_tip3p) prior to energy minimization consisting of 25,000 steps of steepest descent with positional constraints placed on the protein backbone atoms. This was followed by an equilibration run with the protein backbone atoms and 2NI molecules restrained, using the NTP ensemble with a constant 1 atm pressure (Berendsen barostat) and a pressure relaxation time of 2 ps and a target temperature of 298 K (Langevin thermostat). SHAKE constraints were employed for all bonds involving hydrogen atoms, and the non-bonded distance cutoff was set to 12 Å. Coordinate frames of the trajectory were captured every 1000 steps for a total of 500 frames over 1 ns. The same parameters were used for the unrestrained MD production run over 500 ns, where 50,000 frames were captured every 5000 steps. Two additional independent production runs were performed using AMBER22 with these same conditions to confirm convergence using RMSD overtime and per residue B-factor comparison.

Trajectory and associated data analyses were carried out using the CPPTRAJ program from the AMBER20 suite and in-house Python scripts. For cluster analysis in CPPTRAJ, all frames from one simulation were first aligned by the protein backbone, and clustered based on the RMSD of the 2NI molecule bound to chain A of the homodimer, as well as residues C357, N311, and A200. The Hierarchical Agglomerative Clustering algorithm was used on every 10^th^ frame of the trajectory to obtain representative and averaged structures for each cluster, with an average minimum distance cut-off between clusters set as 1 Å.

### Statistics and reproducibility

Statistics for MD simulations and structural analysis using X-ray crystallography and small X-ray light angle scattering was performed using their data analysis software as described in the respective sections. Multiple data sets were collected for the X-ray crystallography (*n* = 4), x-ray light angle scattering (*n* = 28), MD simulations (*n* = 3), DSF (*n* = 3), and enzyme kinetics (*n* = 2) to ensure reproducibility.

## Supplementary information

Supplementary Information

Description of Additional Supplementary Files

Supplementary Data 1

Supplementary Data 2

Supplementary Data 3

nr-reporting-summary

## Data Availability

The model coordinates for the protein structures are publicly available for download from the Protein Data Bank under accession codes: 9AZG, 9AZH, 9B01, and 9B02. The starting and final coordinates from the MD simulation are available as supplementary files (Supplementary Data 1, 2, respectively). The SEC-SAXS data is available from the Small Angle Scattering Biological Data Bank (https://www.sasbdb.org/) under the accession code SASDVG6, and raw data for the graphs are provided in Supplementary Data 3. The plasmid DNA for NnhA-T2I-G14D-K73R is available from Addgene (ID: 231043). All other data supporting the findings are available from the corresponding authors upon request.

## References

[1] Okami, Y., Maeda, K. & Umezawa, H. Studies on antibiotic actinomycetes. VII. Azomycin-producing strain resembling to Nocardia mesenterica. *J. Antibiot.* **7**, 53–56 (1954). 13174450

[2] Shoji, J. et al. Isolation of azomycin from *Pseudomonas fluorescens*. *J. Antibiot.* **42**, 1513–1514 (1989). 10.7164/antibiotics.42.1513 2509407

[3] Qu, Y. & Spain, J. C. Catabolic pathway for 2-nitroimidazole involves a novel nitrohydrolase that also confers drug resistance. *Environ. Microbiol.* **13**, 1010–1017 (2011). 21244596 10.1111/j.1462-2920.2010.02406.x

[4] Goldstein, B. P. et al. The mechanism of action of nitro-heterocyclic antimicrobial drugs. Metabolic activation by micro-organisms. *J. Gen. Microbiol.* **100**, 283–298 (1977). 330810 10.1099/00221287-100-2-283

[5] Nunn, A., Linder, K. & Strauss, H. W. Nitroimidazoles and imaging hypoxia. *Eur. J. Nucl. Med.* **22**, 265–280 (1995). 7789400 10.1007/BF01081524

[6] Ballinger, J. R. Imaging hypoxia in tumors. *Semin. Nucl. Med.* **31**, 321–329 (2001). 11710774 10.1053/snuc.2001.26191

[7] Li, Z. & Chu, T. Recent advances on radionuclide labeled hypoxia-imaging agents. *Curr. Pharm. Des.* **18**, 1084–1097 (2012). 22272826 10.2174/138161212799315849

[8] Rashed, F. Bin et al. Cellular mechanism of action of 2-nitroimidazoles as hypoxia-selective therapeutic agents. *Redox Biol.* **52**, 102300 (2022). 35430547 10.1016/j.redox.2022.102300 PMC9038562

[9] Koike, N. et al. 2-Nitroimidazoles induce mitochondrial stress and ferroptosis in glioma stem cells residing in a hypoxic niche. *Commun. Biol.* **3**, 1–13 (2020). 32807853 10.1038/s42003-020-01165-z PMC7431527

[10] Melo, T., Ballinger, J. R. & Rauth, A. M. Role of NADPH:cytochrome P450 reductase in the hypoxic accumulation and metabolism of BRU59-21, a technetium-99m-nitroimidazole for imaging tumor hypoxia. *Biochem. Pharm.* **60**, 625–634 (2000). 10927020 10.1016/s0006-2952(00)00373-7

[11] Edwards, D. I. Nitroimidazole drugs-action and resistance mechanisms I. Mechanism of action. *J. Antimicrob. Chemother.* **31**, 9–20 (1993). 8444678 10.1093/jac/31.1.9

[12] Tocher, J. H. & Edwards, D. I. Evidence for the direct interaction of reduced metronidazole derivatives with DNA bases. *Biochem. Pharm.* **48**, 1089–1094 (1994). 7945401 10.1016/0006-2952(94)90144-9

[13] McClelland, R. A., Panicucci, R. & Rauth, A. M. Products of the reductions of 2-nitroimidazoles. *J. Am. Chem. Soc.* **109**, 4308–4314 (1987).

[14] Ju, K.-S. & Parales, R. E. Nitroaromatic compounds, from synthesis to biodegradation. *Microbiol. Mol. Biol. Rev.* **74**, 250 (2010). 20508249 10.1128/MMBR.00006-10 PMC2884413

[15] Weickmann, J. L. & Fahrney, D. E. Arginine deiminase from *Mycoplasma arthritidis*. Evidence for multiple forms. *J. Biol. Chem.* **252**, 2615–2620 (1977). 856796

[16] Ogawa, T., Kimoto, M. & Sasaoka, K. Purification and properties of a new enzyme, NG, NG-dimethylarginine dimethylaminohydrolase, from rat kidney. *J. Biol. Chem.* **264**, 10205–10209 (1989). 2722865

[17] Murray-Rust, J. et al. Structural insights into the hydrolysis of cellular nitric oxide synthase inhibitors by dimethylarginine dimethylaminohydrolase. *Nat. Struct. Biol.* **8**, 679–683 (2001). 11473257 10.1038/90387

[18] Lu, X., Galkin, A., Herzberg, O. & Dunaway-Mariano, D. Arginine deiminase uses an active-site cysteine in nucleophilic catalysis of L-arginine hydrolysis. *J. Am. Chem. Soc.* **126**, 5374–5375 (2004). 15113205 10.1021/ja049543p

[19] Wei, Y., Zhou, H., Sun, Y., He, Y. & Luo, Y. Insight into the catalytic mechanism of arginine deiminase: functional studies on the crucial sites. *Proteins* **66**, 740–750 (2007). 17080455 10.1002/prot.21235

[20] Das, K. et al. Crystal structures of arginine deiminase with covalent reaction intermediates: implications for catalytic mechanism. *Structure* **12**, 657–667 (2004). 15062088 10.1016/j.str.2004.02.017

[21] Li, L. et al. The electrostatic driving force for nucleophilic catalysis in L-arginine deiminase: a combined experimental and theoretical study. *Biochemistry* **47**, 4721–4732 (2008). 18366187 10.1021/bi7023496

[22] Johnson, C. M. & Fast, W. On the kinetic mechanism of dimethylarginine dimethylaminohydrolase. *Bioorg. Med. Chem.* **66**, 116816 (2022). 35598478 10.1016/j.bmc.2022.116816

[23] Shirai, H., Blundell, T. L. & Mizuguchi, K. A novel superfamily of enzymes that catalyze the modification of guanidino groups. *Trends Biochem. Sci.* **26**, 465–468 (2001). 11504612 10.1016/s0968-0004(01)01906-5

[24] Shirai, H., Mokrab, Y. & Mizuguchi, K. The guanidino-group modifying enzymes: structural basis for their diversity and commonality. *Proteins* **64**, 1010–1023 (2006). 16779844 10.1002/prot.20863

[25] Kanehisa, M., Furumichi, M., Tanabe, M., Sato, Y. & Morishima, K. KEGG: new perspectives on genomes, pathways, diseases and drugs. *Nucleic Acids Res.* **45**, D353–D361 (2017). 27899662 10.1093/nar/gkw1092 PMC5210567

[26] Shirai, H. & Mizuguchi, K. Prediction of the structure and function of AstA and AstB, the first two enzymes of the arginine succinyltransferase pathway of arginine catabolism. *FEBS Lett.* **555**, 505–510 (2003). 14675764 10.1016/s0014-5793(03)01314-0

[27] Gerlt, J. A. et al. Enzyme function initiative-enzyme similarity tool (EFI-EST): a web tool for generating protein sequence similarity networks. *Biochim. Biophys. Acta* **1854**, 1019–1037 (2015). 25900361 10.1016/j.bbapap.2015.04.015 PMC4457552

[28] Ahmed, F. H. et al. Sequence-structure-function classification of a catalytically diverse oxidoreductase superfamily in mycobacteria. *J. Mol. Biol.* **427**, 3554–3571 (2015). 26434506 10.1016/j.jmb.2015.09.021

[29] Atkinson, H. J., Morris, J. H., Ferrin, T. E. & Babbitt, P. C. Using sequence similarity networks for visualization of relationships across diverse protein superfamilies. *PLoS ONE* **4**, e4345 (2009). 19190775 10.1371/journal.pone.0004345 PMC2631154

[30] Uberto, R. & Moomaw, E. W. Protein similarity networks reveal relationships among sequence, structure, and function within the cupin superfamily. *PLoS ONE* **8**, e74477 (2013). 24040257 10.1371/journal.pone.0074477 PMC3765361

[31] O’Leary, N. A. et al. Reference sequence (RefSeq) database at NCBI: current status, taxonomic expansion, and functional annotation. *Nucleic Acids Res.* **44**, D733–D745 (2016). 26553804 10.1093/nar/gkv1189 PMC4702849

[32] Jackson, C. J. et al. 300-fold increase in production of the Zn2+-dependent dechlorinase trzN in soluble form via apoenzyme stabilization. *Appl. Environ. Microbiol.* **80**, 4003–4011 (2014). 24771025 10.1128/AEM.00916-14 PMC4054219

[33] Watson, M., Liu, J. W. & Ollis, D. Directed evolution of trimethoprim resistance in *Escherichia coli*. *FEBS J.* **274**, 2661–2671 (2007). 17451440 10.1111/j.1742-4658.2007.05801.x

[34] Hou, C. -F. D. et al. Insights into an evolutionary strategy leading to antibiotic resistance. *Sci. Rep.* **7**, 40357 (2017). 10.1038/srep40357 PMC5225480 28074907

[35] Hur, S. & Bruice, T. C. From the cover: the near attack conformation approach to the study of the chorismate to prephenate reaction. *Proc. Natl Acad. Sci. USA* **100**, 12015 (2003). 14523243 10.1073/pnas.1534873100 PMC218705

[36] Humm, A., Fritsche, E., Steinbacher, S. & Huber, R. Crystal structure and mechanism of human L-arginine:glycine amidinotransferase: a mitochondrial enzyme involved in creatine biosynthesis. *EMBO J.* **16**, 3373–3385 (1997). 9218780 10.1093/emboj/16.12.3373 PMC1169963

[37] Krissinel, E. & Henrick, K. Inference of macromolecular assemblies from crystalline state. *J. Mol. Biol.* **372**, 774–797 (2007). 17681537 10.1016/j.jmb.2007.05.022

[38] Trewhella, J. Recent advances in small-angle scattering and its expanding impact in structural biology. *Structure* **30**, 15–23 (2022). 34995477 10.1016/j.str.2021.09.008

[39] Hura, G. L. et al. Small angle X-ray scattering-assisted protein structure prediction in CASP13 and emergence of solution structure differences. *Proteins* **87**, 1298 (2019). 31589784 10.1002/prot.25827 PMC6851496

[40] van Kempen, M. et al. Fast and accurate protein structure search with Foldseek. *Nat. Biotechnol.* **42**, 243–246 (2023). 37156916 10.1038/s41587-023-01773-0 PMC10869269

[41] Xu, J. & Zhang, Y. How significant is a protein structure similarity with TM-score = 0.5? *Bioinformatics* **26**, 889 (2010). 20164152 10.1093/bioinformatics/btq066 PMC2913670

[42] Leiper, J. et al. Disruption of methylarginine metabolism impairs vascular homeostasis. *Nat. Med.* **13**, 198–203 (2007). 17273169 10.1038/nm1543

[43] Galkin, A., Lu, X., Dunaway-Mariano, D. & Herzberg, O. Crystal structures representing the Michaelis complex and the thiouronium reaction intermediate of *Pseudomonas aeruginosa* arginine deiminase. *J. Biol. Chem.* **280**, 34080–34087 (2005). 16091358 10.1074/jbc.M505471200

[44] Galkin, A. et al. Structural insight into arginine degradation by arginine deiminase, an antibacterial and parasite drug target. *J. Biol. Chem.* **279**, 14001–14008 (2004). 14701825 10.1074/jbc.M313410200

[45] Gallego, P. et al. Structural characterization of the enzymes composing the arginine deiminase pathway in *Mycoplasma penetrans*. *PLoS ONE* **7**, e47886 (2012). 23082227 10.1371/journal.pone.0047886 PMC3474736

[46] Henningham, A. et al. Structure-informed design of an enzymatically inactive vaccine component for group A Streptococcus. *mBio* **4**, e00509-13 (2013). 10.1128/mBio.00509-13 PMC3735194 23919999

[47] Ravindranath, P. A., Forli, S., Goodsell, D. S., Olson, A. J. & Sanner, M. F. AutoDockFR: advances in protein-ligand docking with explicitly specified binding site flexibility. *PLoS Comput. Biol.* **11**, 1004586 (2015). 10.1371/journal.pcbi.1004586 PMC4667975 26629955

[48] Lee, B. M. et al. Predicting nitroimidazole antibiotic resistance mutations in Mycobacterium tuberculosis with protein engineering. *PLoS Pathog.* **16**, e1008287 (2020). 10.1371/journal.ppat.1008287 PMC7032734 32032366

[49] Stone, E. M., Costello, A. L., Tierney, D. L. & Fast, W. Substrate-assisted cysteine deprotonation in the mechanism of dimethylargininase (DDAH) from *Pseudomonas aeruginosa*. *Biochemistry* **45**, 5618–5630 (2006). 16634643 10.1021/bi052595m

[50] Trezza, A., Cicaloni, V., Pettini, F. & Spiga, O. in *Cancer-Leading Proteases: Structures, Functions, and Inhibition* (ed. Gupta, S. P.) Ch. 2 (Elsevier Science, 2020)

[51] Oanca, G., Asadi, M., Saha, A., Ramachandran, B. & Warshel, A. Exploring the catalytic reaction of cysteine proteases. *J. Phys. Chem. B* **124**, 11349–11356 (2020). 33264018 10.1021/acs.jpcb.0c08192

[52] Neylon, C. et al. Interaction of the Escherichia coli replication terminator protein (Tus) with DNA: a model derived from DNA-binding studies of mutant proteins by surface plasmon resonance. Biochemistry **39**, 11989–11999 (2000). 10.1021/bi001174w 11009613

[53] Alfaro-Chávez, A. L., Liu, J. W., Stevenson, B. J., Goldman, A. & Ollis, D. L. Evolving a lipase for hydrolysis of natural triglycerides along with enhanced tolerance towards a protease and surfactants. Protein Engineering, Design and Selection **32**, 129–143 (2019). 10.1093/protein/gzz023 31504920

[54] Seabrook, S. A. & Newman, J. High-throughput thermal scanning for protein stability: making a good technique more robust. *ACS Comb. Sci.* **15**, 387–392 (2013). 23710551 10.1021/co400013v

[55] Rosa, N. et al. Meltdown: a tool to help in the interpretation of thermal melt curves acquired by differential scanning fluorimetry. *SLAS Discov.* **20**, 898–905 (2015). 10.1177/1087057115584059 25918038

[56] Panjikar, S., Parthasarathy, V., Lamzin, V. S., Weiss, M. S. & Tucker, P. A. Auto-rickshaw: an automated crystal structure determination platform as an efficient tool for the validation of an X-ray diffraction experiment. *Acta Crystallogr. D. Biol. Crystallogr.* **61**, 449–457 (2005). 15805600 10.1107/S0907444905001307

[57] McCoy, A. J. et al. Phaser crystallographic software. *J. Appl. Crystallogr.* **40**, 658–674 (2007). 19461840 10.1107/S0021889807021206 PMC2483472

[58] Kabsch, W. XDS. *Acta Crystallogr. D. Struct. Biol.* **66**, 125–132 (2010). 10.1107/S0907444909047337 PMC2815665 20124692

[59] Emsley, P., Lohkamp, B., Scott, W. G. & Cowtan, K. Features and development of Coot. *Acta Crystallogr. D. Biol. Crystallogr.* **66**, 486–501 (2010). 20383002 10.1107/S0907444910007493 PMC2852313

[60] Murshudov, G. N. et al. REFMAC5 for the refinement of macromolecular crystal structures. *Acta Crystallogr. Sect. D.* **67**, 355–367 (2011). 21460454 10.1107/S0907444911001314 PMC3069751

[61] Shannon, P. et al. Cytoscape: a software environment for integrated models of biomolecular interaction networks. *Genome Res.* **13**, 2498–2504 (2003). 14597658 10.1101/gr.1239303 PMC403769

[62] Fu, L., Niu, B., Zhu, Z., Wu, S. & Li, W. CD-HIT: accelerated for clustering the next-generation sequencing data. *Bioinformatics* **28**, 3150–3152 (2012). 23060610 10.1093/bioinformatics/bts565 PMC3516142

[63] Okonechnikov, K. et al. Unipro UGENE: a unified bioinformatics toolkit. *Bioinformatics* **28**, 1166–1167 (2012). 22368248 10.1093/bioinformatics/bts091

[64] Katoh, K. & Standley, D. M. MAFFT multiple sequence alignment software version 7: improvements in performance and usability. *Mol. Biol. Evol.* **30**, 772–780 (2013). 23329690 10.1093/molbev/mst010 PMC3603318

[65] Price, M. N., Dehal, P. S. & Arkin, A. P. FastTree 2-approximately maximum-likelihood trees for large alignments. *PLoS ONE* **5**, e9490 (2010). 20224823 10.1371/journal.pone.0009490 PMC2835736

[66] Menardo, F. et al. Treemmer: a tool to reduce large phylogenetic datasets with minimal loss of diversity. *BMC Bioinformatics* **19**, 164 (2018). 10.1186/s12859-018-2164-8 PMC5930393 29716518

[67] Minh, B. Q. et al. IQ-TREE 2: new models and efficient methods for phylogenetic inference in the genomic era. *Mol. Biol. Evol.* **37**, 1530–1534 (2020). 32011700 10.1093/molbev/msaa015 PMC7182206

[68] Ryan, T. M. et al. An optimized SEC-SAXS system enabling high X-ray dose for rapid SAXS assessment with correlated UV measurements for biomolecular structure analysis. *J. Appl Crystallogr.* **51**, 97–111 (2018).

[69] Kirby, N. et al. Improved radiation dose efficiency in solution SAXS using a sheath flow sample environment. *Acta Crystallogr. D. Struct. Biol.* **72**, 1254–1266 (2016). 27917826 10.1107/S2059798316017174 PMC5137223

[70] Hopkins, J. B. BioXTAS RAW 2: new developments for a free open-source program for small-angle scattering data reduction and analysis. *J. Appl. Crystallogr.* **57**, 194–208 (2024). 38322719 10.1107/S1600576723011019 PMC10840314

[71] Manalastas-Cantos, K. et al. *ATSAS 3.0*: expanded functionality and new tools for small-angle scattering data analysis. *J. Appl. Crystallogr*. **54**, 343–355 (2021). 10.1107/S1600576720013412 PMC7941305 33833657

[72] Brookes, E., Vachette, P., Rocco, M. & Pérez, J. US-SOMO HPLC-SAXS module: dealing with capillary fouling and extraction of pure component patterns from poorly resolved SEC-SAXS data. *J. Appl. Crystallogr.* **49**, 1827–1841 (2016). 27738419 10.1107/S1600576716011201 PMC5045733

[73] Svergun, D. I. Determination of the regularization parameter in indirect-transform methods using perceptual criteria. *J. Appl. Crystallogr.* **25**, 495–503 (1992).

[74] Petoukhov, M. V., Konarev, P. V., Kikhney, A. G. & Svergun, D. I. ATSAS 2.1 - Towards automated and web-supported small-angle scattering data analysis. *J. Appl. Crystallogr.* **40**, s223–s228 (2007).

[75] Hajizadeh, N. R., Franke, D., Jeffries, C. M. & Svergun, D. I. Consensus Bayesian assessment of protein molecular mass from solution X-ray scattering data. *Sci. Rep.* **8**, 1–13 (2018). 29739979 10.1038/s41598-018-25355-2 PMC5940760

[76] Rambo, R. P. & Tainer, J. A. Accurate assessment of mass, models and resolution by small-angle scattering. *Nature* **496**, 477–481 (2013). 23619693 10.1038/nature12070 PMC3714217

[77] Piiadov, V., Ares de Araújo, E., Oliveira Neto, M., Craievich, A. F. & Polikarpov, I. SAXSMoW 2.0: online calculator of the molecular weight of proteins in dilute solution from experimental SAXS data measured on a relative scale. *Protein Sci.* **28**, 454–463 (2019). 30371978 10.1002/pro.3528 PMC6319763

[78] Svergun, D., Barberato, C. & Koch, M. H. CRYSOL– a program to evaluate X-ray solution scattering of biological macromolecules from atomic coordinates. *J. Appl. Crystallogr.* **28**, 768–773 (1995).

[79] Svergun, D. I. Restoring low resolution structure of biological macromolecules from solution scattering using simulated annealing. *Biophys. J.* **76**, 2879 (1999). 10354416 10.1016/S0006-3495(99)77443-6 PMC1300260

[80] Volkov, V. V. & Svergun, D. I. Uniqueness of ab initio shape determination in small-angle scattering. *J. Appl. Crystallogr.* **36**, 860–864 (2003). 10.1107/S0021889809000338 PMC5023043 27630371

[81] Kim, S. et al. PubChem 2023 update. *Nucleic Acids Res.* **51**, D1373–D1380 (2023). 36305812 10.1093/nar/gkac956 PMC9825602

[82] Morris, G. M. et al. AutoDock4 and AutoDockTools4: automated docking with selective receptor flexibility. *J. Comput. Chem.* **30**, 2785–2791 (2009). 19399780 10.1002/jcc.21256 PMC2760638

[83] Case, D. A. et al. *AMBER 2020* (2020).

[84] Zhang, H., Kim, S. & Im, W. Practical guidance for consensus scoring and force field selection in protein-ligand binding free energy simulations. *J. Chem. Inf. Model* **62**, 6084 (2022). 36399655 10.1021/acs.jcim.2c01115 PMC9772090

[85] Zhuang, N. et al. Crystal structures and biochemical analyses of the bacterial arginine dihydrolase ArgZ suggests a “bond rotation” catalytic mechanism. *J. Biol. Chem.* **295**, 2113–2124 (2020). 31914412 10.1074/jbc.RA119.011752 PMC7029115

[86] Lee, H. & Rhee, S. Structural and mutational analyses of the bifunctional arginine dihydrolase and ornithine cyclodeaminase AgrE from the cyanobacterium Anabaena. *J. Biol. Chem.* **295**, 5751–5760 (2020). 32198136 10.1074/jbc.RA120.012768 PMC7186175

[87] Tocilj, A. et al. Crystal structure of N-succinylarginine dihydrolase AstB, bound to substrate and product, an enzyme from the arginine catabolic pathway of Escherichia coli. *J. Biol. Chem.* **280**, 15800–15808 (2005). 15703173 10.1074/jbc.M413833200

[88] Laskowski, R. A. & Swindells, M. B. LigPlot+: multiple ligand-protein interaction diagrams for drug discovery. *J. Chem. Inf. Model* **51**, 2778–2786 (2011). 21919503 10.1021/ci200227u

