# Supplementary material for: Structural insights into the enzymatic breakdown of azomycin-derived antibiotics by 2-nitroimdazole hydrolase (NnhA)
Source: Commun Biol. 2024 Dec 19;7:1676. doi: 10.1038/s42003-024-07336-6 (PMC11659421; doi:10.1038/s42003-024-07336-6)
Supplement: Supplementary file 1 — Supplementary Information [file 42003_2024_7336_MOESM1_ESM.pdf]

Supplementary Information

Structural insights into the enzymatic breakdown of azomycin-derived antibiotics by 2-nitroimdazole hydrolase (NnhA)

F Hafna Ahmed[a,b]*, Jian-Wei Liu[a], Santana Royan[c], Andrew C Warden[a,b], Lygie Esquirol[a,b], Gunjan Pandey[a], Janet Newman[c,e], Colin Scott[a,b,d], Thomas S Peat[c,e]*

[a] Environment, CSIRO, Canberra, ACT, 2601, Australia

[b] Advanced Engineering Biology Future Science Platform, CSIRO, Canberra, ACT, 2601, Australia

[c] Manufacturing, CSIRO, 343 Royal Parade, Parkville, VIC, 3052, Australia

[d] ARC Centre of Excellence in Synthetic Biology, CSIRO, Canberra, ACT 2601, Australia

[e] BABS, UNSW, Kensington, NSW, 2052, Australia

*Correspondence email: t.peat@unsw.edu.au, hafna.ahmed@csiro.au

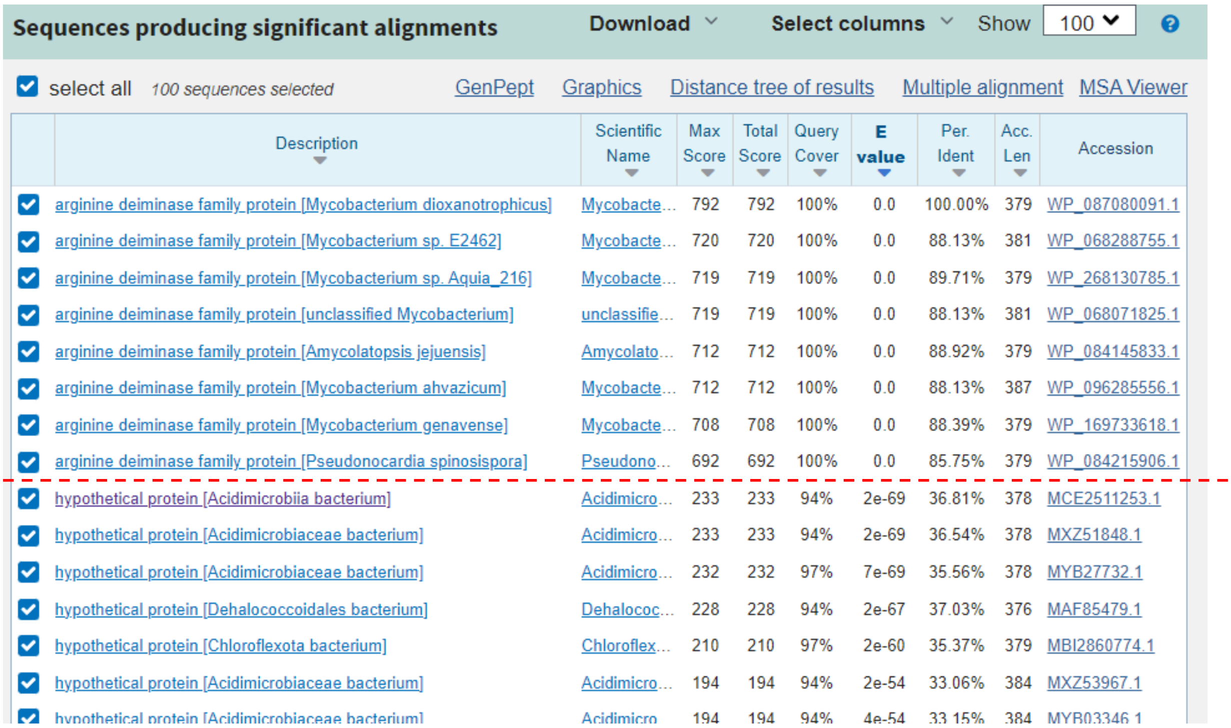

Sequences producing significant alignments | Download | Select columns | Show 100

select all 100 sequences selected | GenPept | Graphics | Distance tree of results | Multiple alignment | MSA Viewer

	Description	Scientific Name	Max Score	Total Score	Query Cover	E value	Per. Ident	Acc. Len	Accession
☑	arginine deiminase family protein [Mycobacterium dioxanotrophicus]	Mycobacte...	792	792	100%	0.0	100.00%	379	WP_087080091.1
☑	arginine deiminase family protein [Mycobacterium sp. E2462]	Mycobacte...	720	720	100%	0.0	88.13%	381	WP_068288755.1
☑	arginine deiminase family protein [Mycobacterium sp. Aquia_216]	Mycobacte...	719	719	100%	0.0	89.71%	379	WP_268130785.1
☑	arginine deiminase family protein [unclassified Mycobacterium]	unclassifie...	719	719	100%	0.0	88.13%	381	WP_068071825.1
☑	arginine deiminase family protein [Amycolatopsis jejuensis]	Amycolato...	712	712	100%	0.0	88.92%	379	WP_084145833.1
☑	arginine deiminase family protein [Mycobacterium ahvazicum]	Mycobacte...	712	712	100%	0.0	88.13%	387	WP_096285556.1
☑	arginine deiminase family protein [Mycobacterium genavense]	Mycobacte...	708	708	100%	0.0	88.39%	379	WP_169733618.1
☑	arginine deiminase family protein [Pseudonocardia spinosispora]	Pseudono...	692	692	100%	0.0	85.75%	379	WP_084215906.1
☑	hypothetical protein [Acidimicrobiia bacterium]	Acidimicro...	233	233	94%	2e-69	36.81%	378	MCE2511253.1
☑	hypothetical protein [Acidimicrobiaceae bacterium]	Acidimicro...	233	233	94%	2e-69	36.54%	378	MXZ51848.1
☑	hypothetical protein [Acidimicrobiaceae bacterium]	Acidimicro...	232	232	97%	7e-69	35.56%	378	MYB27732.1
☑	hypothetical protein [Dehalococcoidales bacterium]	Dehalococ...	228	228	94%	2e-67	37.03%	376	MAF85479.1
☑	hypothetical protein [Chloroflexota bacterium]	Chloroflex...	210	210	97%	2e-60	35.37%	379	MBI2860774.1
☑	hypothetical protein [Acidimicrobiaceae bacterium]	Acidimicro...	194	194	94%	2e-54	33.06%	384	MXZ53967.1
☑	hypothetical protein [Acidimicrobiaceae bacterium]	Acidimicro	194	194	94%	4e-54	33.15%	384	MYB03346.1

Supplementary Figure 1: BLAST search results when queried with NnhA against the NCBI non-redundant protein database. The results show only 8 sequences with >50% sequence identity to NnhA. (Retrieved on 4/08/2023)

Supplementary Table 1: Mutations observed in the most active NnhA variants isolated through library screening.

Variant#	Mutations in isolated NnhA variants
1	T54A
2	V4A, G14D, K231R
3	T2I, G14D, K73R
4	K25R
5	T2I, G14D, A36T, Y74H, D103E, K177R
6	T2I, G14D, K73R

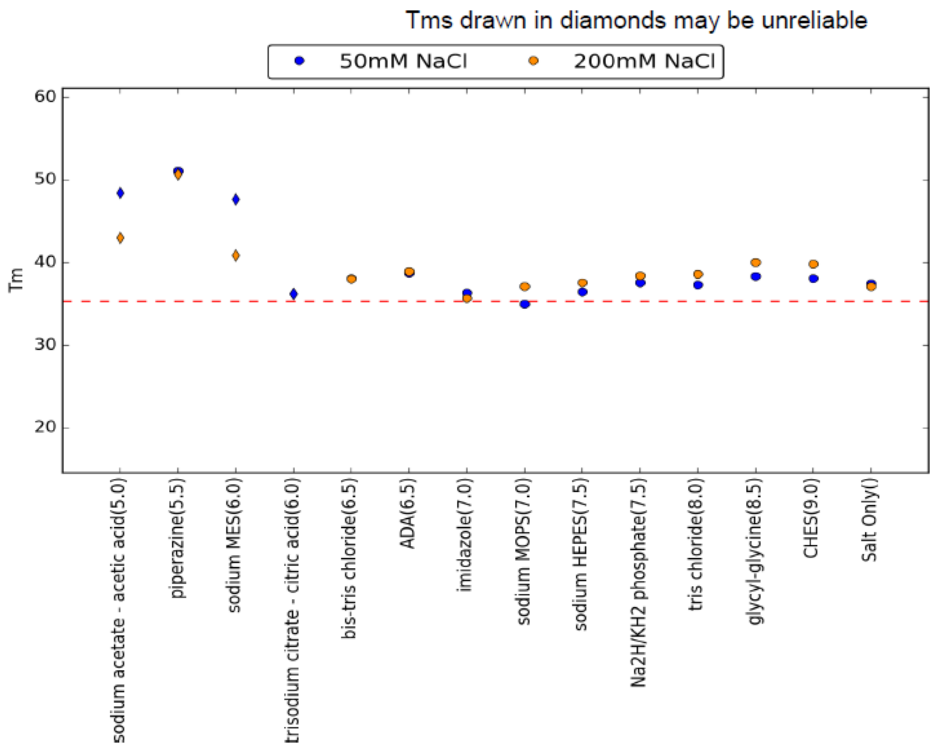

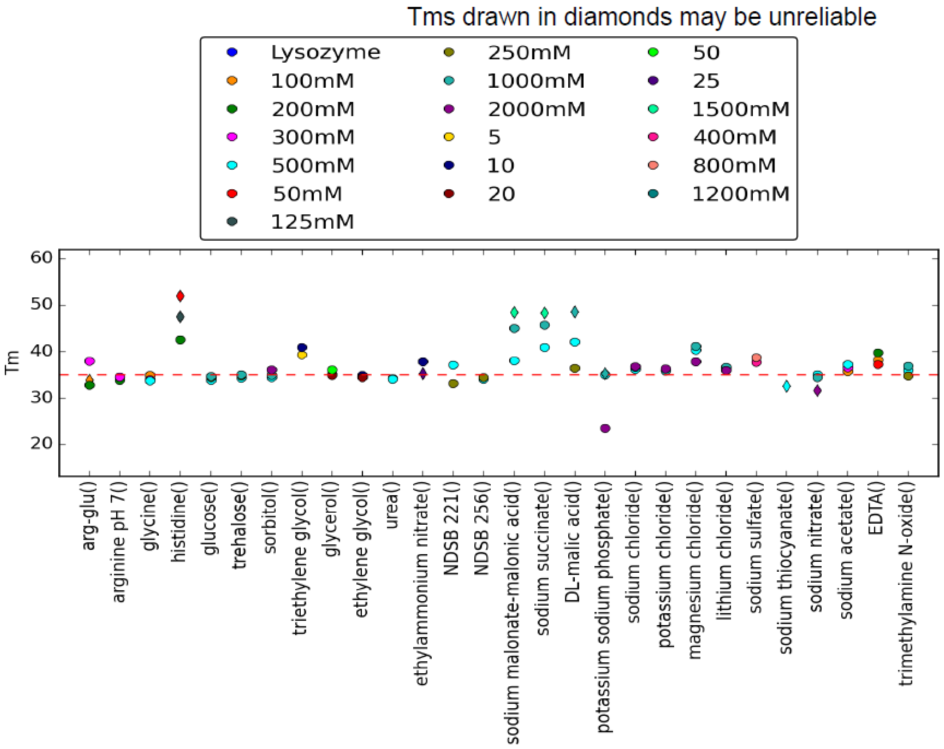

Supplementary Figure 2: summary of DSF screen for buffer conditions that improve protein stability

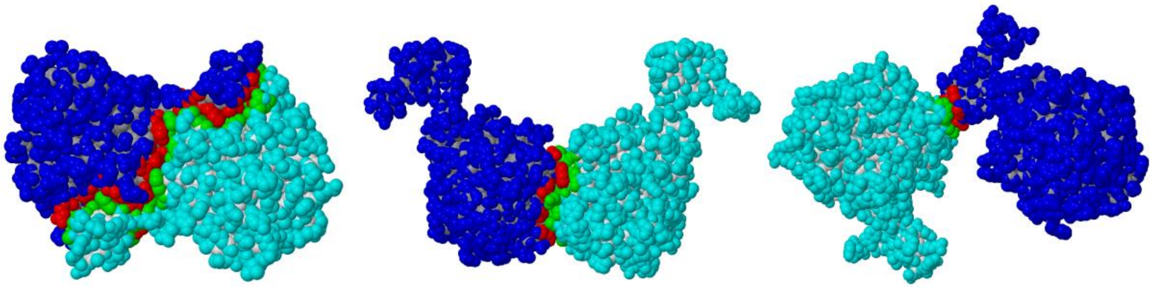

A. Type 1 (dimer formation between protomers) **B.** Type 2 (main dimer-dimer) **C.** Type 3 (minor dimer-dimer, N-term adjacent to dimer partner)

Supplementary Figure 3: Visual representation of the interfaces found in the NnhA hexamer described in table 1

Supplementary Table 2: PDBePISA analysis results for the interfaces within the hexamer. Any interfaces with symmetry mates were not considered.

Type	Interfacing molecules	Interfacing residues (molecule 1)	Interfacing residues (molecule 2)	Interface surface area (Å^2)	ΔG (Kcal/mol)	ΔG P-value*	No. of bonds at interface			CSS**
							H-bonds	Salt bridges	disulfide bonds	
1 (dimer formation)	E-C	86	85	3312.3	-40.6	0.004	36	0	0	1
	B-A	84	85	3250.9	-42.8	0.017	33	0	0	1
	F-D	73	70	2734.5	-32.3	0.074	31	0	0	1
	Average	*81*	*80*	*3099.2*	*-38.5*	*0.032*	*33*	*0*	*0*	*1*
2 (main dimer-dimer)	F-E	24	25	925.1	-6.3	0.353	9	8	0	1
	C-A	24	24	922.3	-8.3	0.249	11	6	0	1
	D-B	18	18	591.6	-5.2	0.581	4	3	0	1
	Average	*22*	*22*	*813*	*-6.6*	*0.394*	*8*	*6*	*0*	*1*
3 (minor dimer-dimer, N-term to protomer adjacent to dimer partner)	E-D	6	5	248	-2.3	0.380	4	4	0	0.1
	F-C	6	6	246.6	-2.5	0.270	4	3	0	0.1
	C-B	6	6	243.9	-2.5	0.303	4	3	0	0.1
	E-A	6	6	242.5	-2.3	0.308	3	4	0	0.1
	D-A	6	6	236.6	-2.0	0.500	4	3	0	0.1
	F-B	3	5	137.5	-0.9	0.447	0	0	0	0.1
	Average	*6*	*6*	*225.9*	*-2.1*	*0.368*	*3*	*3*	*0*	*0.1*

* ΔG P-value is a measure of interface specificity, showing how surprising, in energy terms, the interface is. P>0.5 means that the interface is less hydrophobic than it could be, therefore the interface is likely to be an artefact of crystal packing. P<0.5 indicates interfaces with surprising (higher than would-be-average for given structures) hydrophobicity, implying that the interface surface can be interaction-specific.

** CSS = Complexation Significance Score, where 1 = significant for complex formation, 0.1 = significance borderline, 0 = not significant.

Supplementary Table 3. Small angle x-ray scattering (SAXS) sample details, data collection, analysis, and 3D modelling details for NnhA-T2I-G14D-K73R in solution.

**(*a*) Sample details**	
Organism	*Mycobacterium sp. JS330*
Source	*E. coli* BL21(DE3) expressed (this publication)<br>NnhA
Scattering particle composition	
Protein	UniProt ID F4ZCI3, Residues 1-379 (T2I, G14D, K73R)
Sample environment/configuration	
Solvent composition	25 mM Tris-HCl pH 7.6, 150 mM NaCl, 5% (v/v) glycerol
Sample temperature (°C)	25
In beam sample cell	1 mm quartz capillary with co-flow
Size Exclusion Chromatography SEC-SAS	
Sample injection concentration, mg/ml or g/cm^3	3.50
Sample injection volume, mL	0.05
SEC column type	Superdex S200 5/150 GL (Cytiva)
SEC flowrate, mL/min	0.4
**(*b*) SAS data collection**	
Data acquisition/reduction software	Data was reduced by Fast Azimuthal Integration using Python (PyFAI) with customized algorithms written for the BioSAXS beamline
Source/instrument description or reference	The BioSAXS beamline with Dectris Pilatus3 X 2M detector at the Australian Synchrotron, part of ANSTO
Wavelength (Å)	1.00 (12.4 keV)
Measured *q*-range ($q_{min} – q_{max}$; Å^{-1})	0.0068-0.4748
Method for scaling intensities	Absolute scaling referenced to water
Exposure time, final number of sample frames used for averaging	1 s exposures, 28 frames used for averaging
Additional relevant details	3151 mm sample-to-detector distance. SEC profiles baseline corrected and buffer subtracted in BioXTAS RAW (RAW 2.3.0, Hopkins *et al* 2024, Brookes *et al.* 2016).
**(*c*) SAS-derived structural parameters**	
Methods/Software	BioXTAS RAW (RAW 2.3.0, Hopkins et al 2024), PRIMUS/qt (ATSAS 3.3.0; Manalastas-Cantos et al., 2021), GNOM (Svergun 1992) and AUTORG (Petoukhov et al. 2007).
Guinier Analysis	**NnhA**
I(0) ± s (cm$^{-1;}$ a.u)	0.0117 ± 4.236×10^{-5}
R_g ± s (Å, nm)	45.58 ± 0.2562
min < qR_g < *max* limit<br>(data point range)	0.3077-1.2902 (qmin: 0.00675, qmax: 0.0283)
Linear fit assessment (definition)	0.9847 (r^2)
PDDF/P(r) analysis	**NnhA**
I(0) ± s (cm$^{-1;}$ a.u.)	0.0116 ± 1.69×10^{-5}
R_g ± s (Å)	44.88 ± 0.0502
d_{max} (Å)	126
q-range (Å^{-1}, nm^{-1})	0.0068-0.3998
P(*r*) fit assessment (definition)	1.6155 (χ^2), 0.5596 (Cormap p-value)

**(*d*) Scattering particle size**	
Methods/Software	BioXTAS RAW (RAW 2.3.0, Hopkins *et al* 2024) & PRIMUS/qt (ATSAS 3.3.0; Manalastas-Cantos *et al.*, 2021) for M from Bayesian inference (Hajizadeh *et al.*, 2018), volume of correlation V_c (Rambo & Tainer, 2013), corrected Porod volume (Piiadov *et al.* 2019)
Volume estimates	
Porod volume, V_p (Å^3, nm^3)	372998
Molecular weight (M) estimates (kDa)	
From chemical composition	42.03 (monomer), 84.06 (dimer),<br>168.12 (tetramer), 252.18 (hexamer)
From SAS, concentration independent method<br>(Bayesian Inference with Probability; Confidence Interval with Probability)	242.6 (84.5%); 221.1-372.7 (98.7%)
From SAS, concentration independent method<br>(Volume of correlation)	250.4
Vc (Å^2)	1.19x10^2
Qr (A^3)	3.08x10^4
Q max	0.175422 (8/Rg)
From SAS, concentration independent method<br>(Porod Volume)	259.5
Corrected Vp	3.13x10^5 A^3
**(*e*) Modelling**	
Atomistic modelling method	**Comparison to the crystal structure**
Software	CRYSOL (Svergun *et al.* 1995)
q-range for fit ($q_{min} – q_{max}$; Å^{-1}, nm^{-1})	0.0068-0.3998
Symmetry/anisotropy assumptions	P1/unknown
Number of individual model reconstructions	12
χ^2, CorMap *P*-values for fit	2.293 (χ^2), 1.20×10^{-5} (CorMap p-value)
Shape modelling method	**Dummy-atom modelling and subsequent averaging (Manalastas-Cantos *et al.*, 2021, Svergun 1999, Volkov & Svergun 2003)**
Software	DAMMIN/DAMAVER
q-range for fit ($q_{min} – q_{max}$; Å^{-1}, nm^{-1})	0.0068-0.3998
Symmetry/anisotropy assumptions	P1/unknown
Number of individual model reconstructions	20
χ^2, CorMap *P*-values for fit	1.620 (χ^2), 0.5596 (CorMap p-value)
**(*f*) Data and model deposition**	
SASBDB ID	SASDVG6

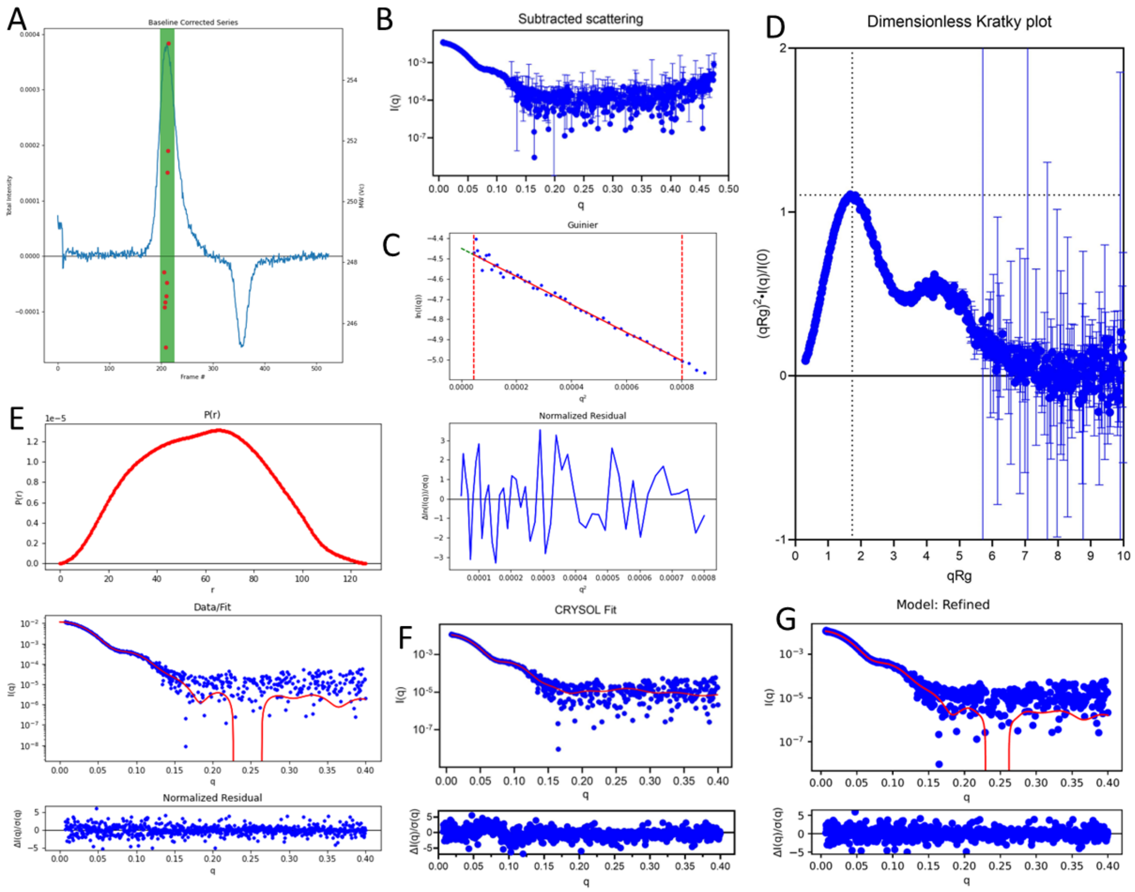

Supplementary Figure 4: SEC-SAXS profile plots for soluble NnhA. **A.** Baseline corrected SEC-SAXS profile for NnhA showing frames used for reduction, with volume of correlation MW plotted on right y-axis .**B.** Log linear plot of subtracted scattering, with error bars. **C.** Guinier plot with normalised residuals.

D. Dimensionless Kratkty plot, with dotted lines at $(qRg)^2 \bullet I(q)/I(0) = 1.104$ and $qRg = \sqrt{3}$ (expected peak for a globular protein). **E.** P(r) function with fit to scattering data and normalised residuals. **F.** CRYSOL fit of NnhA crystal structure and normalised residuals. **G.** Refined DAMMIN model fit and normalised residuals. Error bars represent SEM from 28 frames of data.

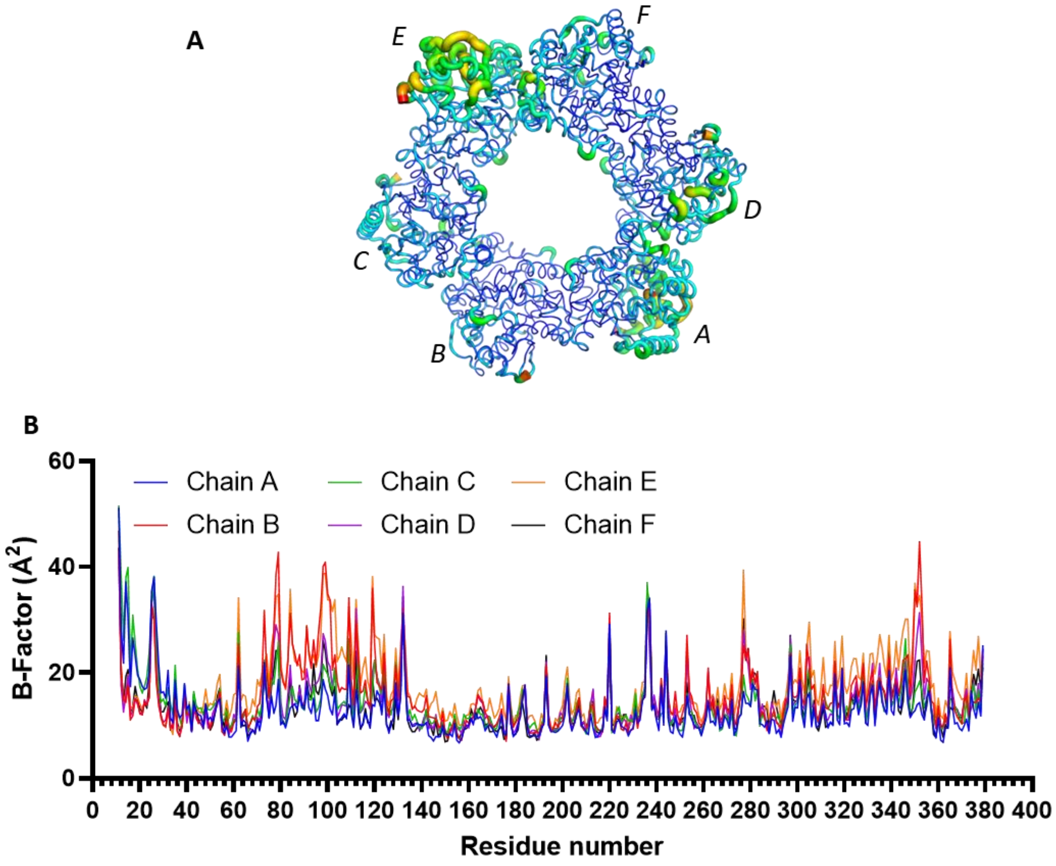

Supplementary Figure 5: Average B-factor per residue in the hexameric crystal structure of NnhA in the P1 space group. **A.** Putty representation of the protein backbone where higher b-factor regions are thicker. Colour gradient is from blue to red for low to high average b-factors, respectively, and the chain ID for the protomers in the hexamer are labelled. **B.** Graphical representation of average B-factors per residue for the 6 protein chains.

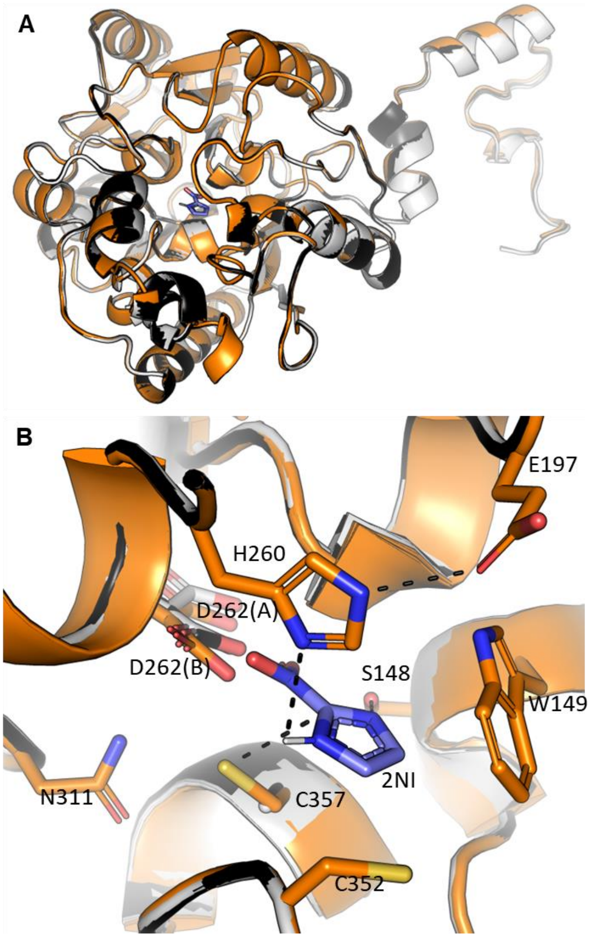

Supplementary Figure 6. Comparison of the structures of the active NnhA-T2I-G14D-K73R (orange) and inactive NnhA-T2I-G14D-K73R-C357A (black and white) variants. **A**. Overall comparison of the structures with NnhA docked in the active site with AutoDockFR. **B.** Comparison of the substrate binding site of the structures.

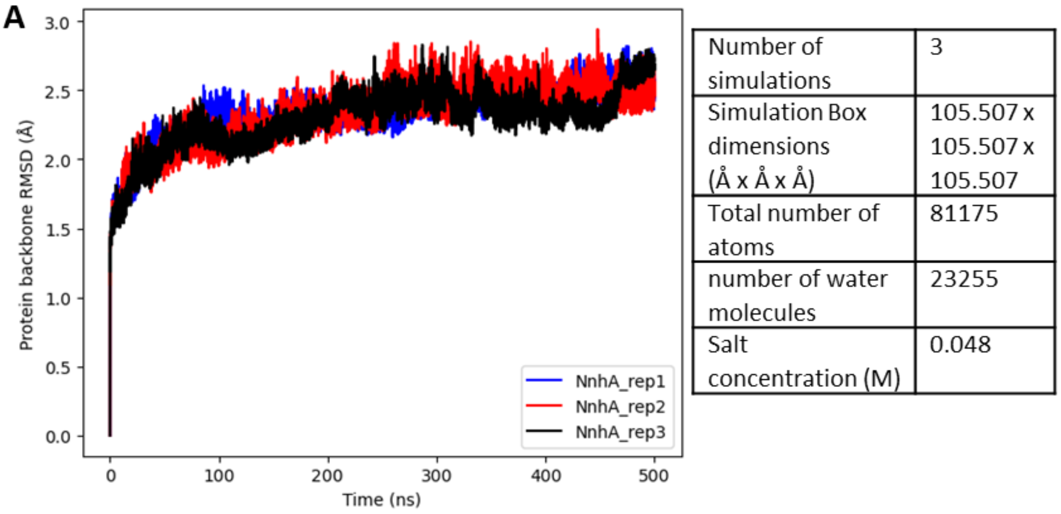

Number of simulations	3
Simulation Box dimensions (Å x Å x Å)	105.507 x 105.507 x 105.507
Total number of atoms	81175
number of water molecules	23255
Salt concentration (M)	0.048

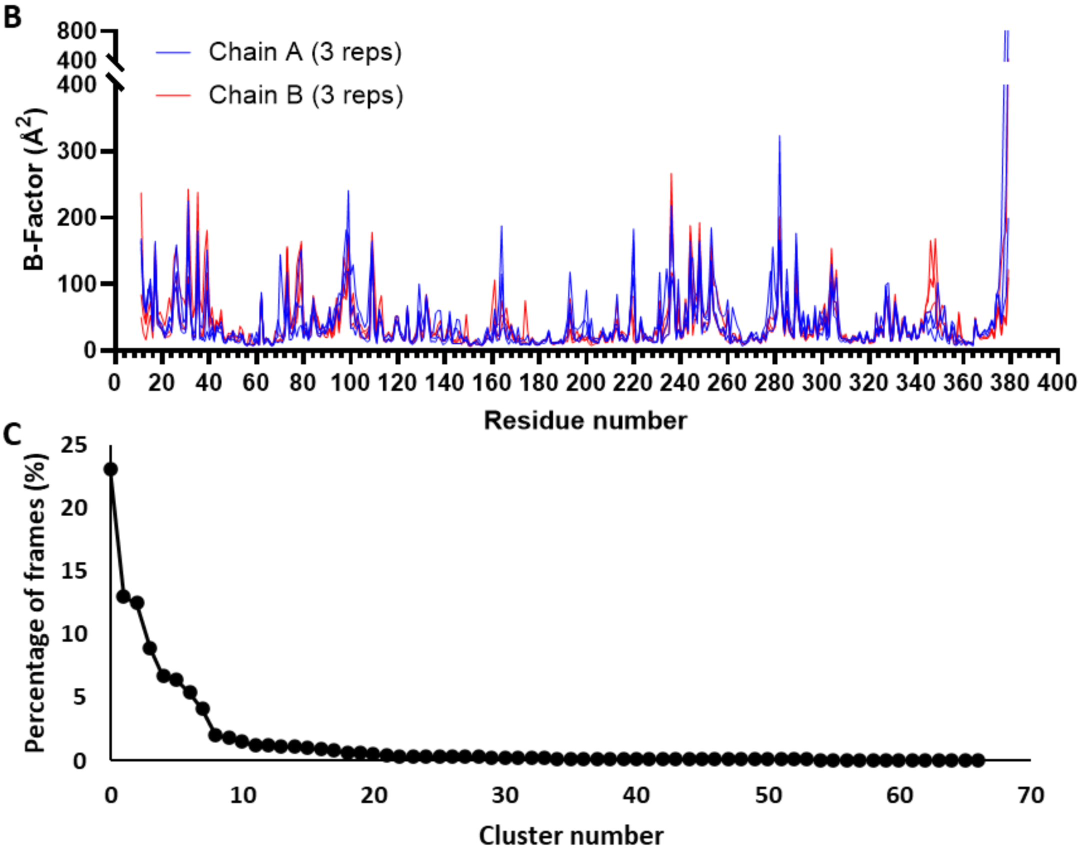

Supplementary Figure 7. A. Stability of the protein backbone during the molecular dynamics (MD) simulation of the NnhA-T2I-G14D-K73R homodimer in n=3 independent simulation runs. **B.** The per residue dynamics of chain A and chain B of the NnhA-T2I-G14D-K73R homodimer during the simulation represented as b-factors, where a separate line represents data from replicate simulations (n=3) for each chain). **C.** Fraction of frames in each cluster obtained when the MD trajectory from rep1 is clustered based on the RMSD of the 2NI molecule in chain A as well as the active site residues C357, N311 and A200.

#Cluster	Frames	Frac	AvgDist	Stdev	Centroid	AvgCDist
**0**	11547	0.231	0.795	0.147	17068	1.967
**1**	6491	0.13	0.761	0.168	8056	2.117
**2**	6236	0.125	0.603	0.127	45511	2.116
**3**	4453	0.089	0.819	0.153	38749	1.829
**4**	3354	0.067	0.65	0.128	307	2.241
**5**	3207	0.064	0.781	0.146	25168	2.032
**6**	2680	0.054	0.79	0.152	30992	1.972
**7**	2034	0.041	0.759	0.166	3681	2.092
**8**	989	0.02	0.703	0.149	35869	1.885
**9**	889	0.018	0.717	0.133	37511	2.289
**10**	739	0.015	0.797	0.163	34022	1.949
**11**	595	0.012	0.816	0.154	6353	2.061
**12**	581	0.012	0.705	0.117	32929	1.97
**13**	573	0.011	0.778	0.188	17508	2.199
**14**	564	0.011	0.716	0.157	42249	1.83
**15**	524	0.01	0.66	0.156	36498	1.867

Supplementary Table 4: Summary data for the first 15 clusters identified from the MD simulation (rep1) of the 2NI bound NnhA-T2I-G14D-K73R homodimer. The most plausible binding mode was cluster 1.

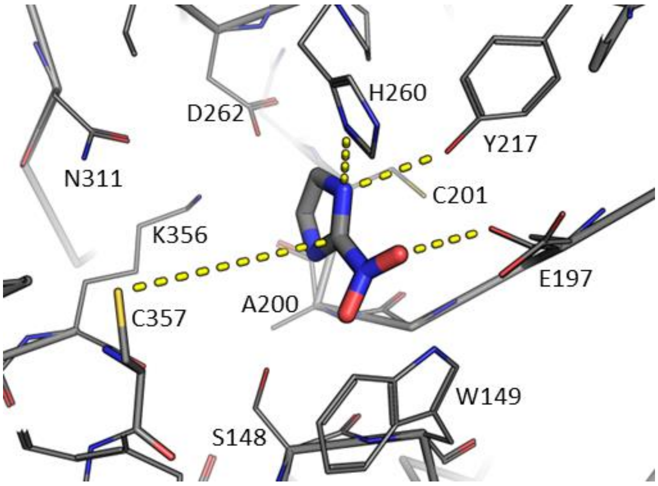

Supplementary Figure 8. 2NI binding pose in the representative structure from the most populated cluster identified from the cluster analysis.

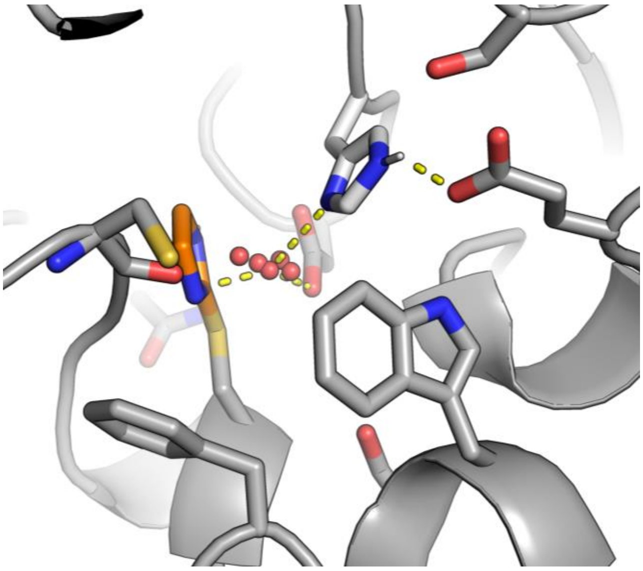

Supplementary Figure 9. Imidazole (orange) covalently docked to C357 of Chain A of the structure NnhA-T2I-G14D-K73R (H32), to demonstrate the planar covalent intermediate formed in the reaction. Water molecules with clear density seen in the experimental data for Chains B and D of the crystal structure within hydrogen bonding distance to H260 and D262 are shown in red, as well as the equivalent water molecules from Chain A of the other 3 structures reported in this study.

References:

Brookes, E., Vachette, P., Rocco, M., & Perez, J. (2016). US-SOMO HPLC-SAXS module: dealing with capillary fouling and extraction of pure component patterns from poorly resolved SEC-SAXS data. *Journal of applied crystallography*, *49*(5), 1827-1841. https://doi.org/doi:10.1107/S1600576716011201

Hajizadeh, N. R., Franke, D., Jeffries, C. M., & Svergun, D. I. (2018). Consensus Bayesian assessment of protein molecular mass from solution X-ray scattering data. *Scientific Reports*, *8*(1), 7204-7204. https://doi.org/10.1038/s41598-018-25355-2

Hopkins, J. (2024). BioXTAS RAW 2: new developments for a free open-source program for small-angle scattering data reduction and analysis. *Journal of applied crystallography*, *57*(1), 194-208. https://doi.org/doi:10.1107/S1600576723011019

Manalastas-Cantos, K., Konarev, P. V., Hajizadeh, N. R., Kikhney, A. G., Petoukhov, M. V., Molodenskiy, D. S., Panjkovich, A., Mertens, H. D. T., Gruzinov, A., Borges, C., Jeffries, C. M., Svergun, D. I., & Franke, D. (2021). ATSAS 3.0: expanded functionality and new tools for small-angle scattering data analysis. *Journal of applied crystallography*, *54*(1), 343-355. https://doi.org/doi:10.1107/S1600576720013412

Petoukhov, M. V., Konarev, P. V., Kikhney, A. G., & Svergun, D. I. (2007). ATSAS 2.1 - towards automated and web-supported small-angle scattering data analysis. *Journal of applied crystallography*, *40*(s1), s223-s228. https://doi.org/doi:10.1107/S0021889807002853

Piiadov, V., Ares de Araújo, E., Oliveira Neto, M., Craievich, A. F., & Polikarpov, I. (2019). SAXSMoW 2.0: Online calculator of the molecular weight of proteins in dilute solution from experimental SAXS data measured on a relative scale. *Protein Sci*, *28*(2), 454-463. https://doi.org/10.1002/pro.3528

Rambo, R. P., & Tainer, J. A. (2013). Accurate assessment of mass, models and resolution by small-angle scattering. *Nature*, *496*(7446), 477-481. https://doi.org/10.1038/nature12070

Svergun, D. I. (1992). Determination of the regularization parameter in indirect-transform methods using perceptual criteria. *Journal of applied crystallography*, *25*(4), 495-503. https://doi.org/10.1107/s0021889892001663

Svergun, D., Barberato, C., & Koch, M. H. J. (1995). CRYSOL - a program to evaluate X-ray solution scattering of biological macromolecules from atomic coordinates. *Journal of applied crystallography*, *28*(6), 768-773. https://doi.org/doi:10.1107/S0021889895007047

Svergun, D. I. (1999). Restoring low resolution structure of biological macromolecules from solution scattering using simulated annealing. *Biophysical Journal*, *76*(6), 2879-2886. https://doi.org/https://doi.org/10.1016/S0006-3495(99)77443-6

Volkov, V. V., & Svergun, D. I. (2003). Uniqueness of ab initio shape determination in small-angle scattering. *Journal of applied crystallography*, *36*(3 Part 1), 860-864. https://doi.org/doi:10.1107/S0021889803000268
