# Supplementary material for: Structural insights into the enzymatic breakdown of azomycin-derived antibiotics by 2-nitroimdazole hydrolase (NnhA)
Source: Commun Biol. 2024 Dec 19;7:1676. doi: 10.1038/s42003-024-07336-6 (PMC11659421; doi:10.1038/s42003-024-07336-6)
Supplement: Supplementary file 2 — Description of Additional Supplementary Files [file 42003_2024_7336_MOESM2_ESM.pdf]

Description of Additional Supplementary Files

File name: Supplementary Data 1
Description: Starting system for MD simulations

File name: Supplementary Data 2
Description: Best ligand binding pose (Chain A) from the MD simulations described in the text.

File name: Supplementary Data 3
Description: Data from BIO_SAXS experiments used for plots in main Figure 4 (panel F) and Supplementary Figure 4.
