# Supplementary material for: Structural insights into the enzymatic breakdown of azomycin-derived antibiotics by 2-nitroimdazole hydrolase (NnhA)
Source: Commun Biol. 2024 Dec 19;7:1676. doi: 10.1038/s42003-024-07336-6 (PMC11659421; doi:10.1038/s42003-024-07336-6)
Supplement: Supplementary file 6 — nr-reporting-summary [file 42003_2024_7336_MOESM6_ESM.pdf]

Corresponding author(s): Hafna Ahmed, Tom Peat

Last updated by author(s): Nov 13, 2024

Reporting Summary

Nature Portfolio wishes to improve the reproducibility of the work that we publish. This form provides structure for consistency and transparency in reporting. For further information on Nature Portfolio policies, see our Editorial Policies and the Editorial Policy Checklist.

Statistics

For all statistical analyses, confirm that the following items are present in the figure legend, table legend, main text, or Methods section.

n/a	Confirmed	
☐	☒	The exact sample size (*n*) for each experimental group/condition, given as a discrete number and unit of measurement
☐	☒	A statement on whether measurements were taken from distinct samples or whether the same sample was measured repeatedly
☒	☐	The statistical test(s) used AND whether they are one- or two-sided<br>*Only common tests should be described solely by name; describe more complex techniques in the Methods section.*
☒	☐	A description of all covariates tested
☒	☐	A description of any assumptions or corrections, such as tests of normality and adjustment for multiple comparisons
☐	☒	A full description of the statistical parameters including central tendency (e.g. means) or other basic estimates (e.g. regression coefficient) AND variation (e.g. standard deviation) or associated estimates of uncertainty (e.g. confidence intervals)
☒	☐	For null hypothesis testing, the test statistic (e.g. *F*, *t*, *r*) with confidence intervals, effect sizes, degrees of freedom and *P* value noted<br>*Give P values as exact values whenever suitable.*
☒	☐	For Bayesian analysis, information on the choice of priors and Markov chain Monte Carlo settings
☒	☐	For hierarchical and complex designs, identification of the appropriate level for tests and full reporting of outcomes
☒	☐	Estimates of effect sizes (e.g. Cohen's *d*, Pearson's *r*), indicating how they were calculated

Our web collection on statistics for biologists contains articles on many of the points above.

Software and code

Policy information about availability of computer code

Data collection	The free-use Enzyme-Function-Initiative Sequence Similarity Tool (EFI_EST) was used via the provided web-server to retrieve protein sequences and generate Sequence Similarity Networks.
Data analysis	CD-Hit, Unipro UGENE v. 43.0, FastTree2.0, TREEMMER v0.3, IQ-Tree v1.6.12, AutodockFR, AutoDockTools, AutoGridFR, AMBER20 suite, CPPTRAJ, AutoRickshaw, XDS, Phaser, REFMAC, Coot, Pymol and BioXTAS-RAW version 2.3.0 are available for download from their respective websites for free-use or with an appropriate license as provided by their authors.

For manuscripts utilizing custom algorithms or software that are central to the research but not yet described in published literature, software must be made available to editors and reviewers. We strongly encourage code deposition in a community repository (e.g. GitHub). See the Nature Portfolio guidelines for submitting code & software for further information.

Data

Policy information about availability of data

All manuscripts must include a data availability statement. This statement should provide the following information, where applicable:

- Accession codes, unique identifiers, or web links for publicly available datasets
- A description of any restrictions on data availability
- For clinical datasets or third party data, please ensure that the statement adheres to our policy

The model coordinates for the protein structures have been deposited to the Protein Data Bank under accession codes: 9AZG, 9AZH, 9B01, and 9B02. The SEC-SAXS

data have been deposited to the Small Angle Scattering Biological Data Bank under the accession code SASDVG6. All other data supporting the findings are available from the corresponding authors upon request.

Research involving human participants, their data, or biological material

Policy information about studies with human participants or human data. See also policy information about sex, gender (identity/presentation), and sexual orientation and race, ethnicity and racism.

Reporting on sex and gender	n/a
Reporting on race, ethnicity, or other socially relevant groupings	n/a
Population characteristics	n/a
Recruitment	n/a
Ethics oversight	n/a

Note that full information on the approval of the study protocol must also be provided in the manuscript.

Field-specific reporting

Please select the one below that is the best fit for your research. If you are not sure, read the appropriate sections before making your selection.

☒ Life sciences ☐ Behavioural & social sciences ☐ Ecological, evolutionary & environmental sciences

For a reference copy of the document with all sections, see nature.com/documents/nr-reporting-summary-flat.pdf

Life sciences study design

All studies must disclose on these points even when the disclosure is negative.

Sample size	Sample size was chosen based on the experiment performed and the generally accepted best practice in the field.
Data exclusions	no data were excluded
Replication	All wet lab and computational experiments were performed in replicate and the details are provided in the manuscript.
Randomization	n/a
Blinding	n/a

Reporting for specific materials, systems and methods

We require information from authors about some types of materials, experimental systems and methods used in many studies. Here, indicate whether each material, system or method listed is relevant to your study. If you are not sure if a list item applies to your research, read the appropriate section before selecting a response.

Materials & experimental systems

n/a	Involved in the study	
☒	☐	Antibodies
☒	☐	Eukaryotic cell lines
☒	☐	Palaeontology and archaeology
☒	☐	Animals and other organisms
☒	☐	Clinical data
☐	☒	Dual use research of concern
☒	☐	Plants

Methods

n/a	Involved in the study	
☒	☐	ChIP-seq
☒	☐	Flow cytometry
☒	☐	MRI-based neuroimaging

Dual use research of concern

Policy information about dual use research of concern

Hazards

Could the accidental, deliberate or reckless misuse of agents or technologies generated in the work, or the application of information presented in the manuscript, pose a threat to:

No	Yes	
☐	☒	Public health
☒	☐	National security
☐	☒	Crops and/or livestock
☒	☐	Ecosystems
☒	☐	Any other significant area

Hazards: The described enzyme is able to degrade a class of antibiotics and render them ineffective, therefore could reduce the potency of antibiotics used for treatments if used deliberately for that purpose. However, the antibiotics is currently only used to treat TB, and more effective drugs are available against TB, so the risk is very low.

For examples of agents subject to oversight, see the United States Government Policy for Institutional Oversight of Life Sciences Dual Use Research of Concern.

Experiments of concern

Does the work involve any of these experiments of concern:

No	Yes	
☒	☐	Demonstrate how to render a vaccine ineffective
☐	☒	Confer resistance to therapeutically useful antibiotics or antiviral agents
☒	☐	Enhance the virulence of a pathogen or render a nonpathogen virulent
☒	☐	Increase transmissibility of a pathogen
☒	☐	Alter the host range of a pathogen
☒	☐	Enable evasion of diagnostic/detection modalities
☒	☐	Enable the weaponization of a biological agent or toxin
☒	☐	Any other potentially harmful combination of experiments and agents

Precautions and benefits

Biosecurity precautions: The study was performed in a PC2 containment facility

Biosecurity oversight: n/a

Benefits: This work describes how soil bacteria degrade antibiotics, helping the understanding of the natural resistance mechanisms against nitroimidazole based antibiotics.

Communication benefits: Understanding the mechanisms involved in antibiotic resistance is required to overcome the emergence of resistance and find alternative modifications to current drugs.

Plants

Seed stocks: n/a

Novel plant genotypes: n/a

Authentication: n/a
